# Genome‐Wide Protein Interaction Analysis in Parasitic Gyrodactylus Flatworms–Fish Hosts System and Drug Target Identification

**DOI:** 10.1002/advs.202514618

**Published:** 2025-09-29

**Authors:** Dong Zhang, Jie‐Mei Zhao, Chuan‐Yu Xiang, Yi‐Wen Ma, Hong‐Peng Lei, Yu‐Ying Shi, Shun Zhou, Xiaofei Zeng, Jinsong Chen, Fei Liu, Benhe Zeng, Rui Song, Ye Hu, Feng Zhang, Xiang Liu, Wen‐Xiang Li, Gui‐Tang Wang, Ivan Jakovlić

**Affiliations:** ^1^ State Key Laboratory of Herbage Improvement and Grassland Agro‐ecosystems and College of Ecology Lanzhou University Lanzhou Gansu 730000 China; ^2^ Key Laboratory of Biodiversity and Environment on the Qinghai‐Tibetan Plateau Ministry of Education School of Ecology and Environment Xizang University Lhasa Xizang 850000 China; ^3^ Shenyang Municipal Bureau of Ecology and Environment Shenyang Liaoning 110000 China; ^4^ State Key Laboratory of Tropical Crop Breeding Shenzhen Branch Guangdong Laboratory for Lingnan Modern Agriculture Genome Analysis Laboratory of the Ministry of Agriculture and Rural Affairs Agricultural Genomics Institute at Shenzhen Chinese Academy of Agricultural Sciences Shenzhen Guangdong 518120 China; ^5^ Yangtze River Fisheries Research Institute Chinese Academy of Fishery Sciences Wuhan Hubei 430223 China; ^6^ State Key Laboratory of Biocontrol and School of Life Sciences Southern Marine Science and Engineering Guangdong Laboratory (Zhuhai) Guangdong Provincial Key Laboratory for Aquatic Economic Animals Sun Yat‐Sen University Guangzhou 510275 China; ^7^ Institute of Fisheries Sciences Xizang Academy of Agricultural and Animal Husbandry Sciences Lhasa Xizang 850032 China; ^8^ Hunan Fisheries Research Institute and Aquatic Products Seed Stock Station Changsha Hunan 410153 China; ^9^ State Key Laboratory of Breeding Biotechnology and Sustainable Aquaculture Institute of Hydrobiology Chinese Academy of Sciences Wuhan 430072 China

**Keywords:** antagonistic coevolution, cAMP signaling, GPCRs, immune coadaptation, innexins, interactome, PRKACB

## Abstract

The host‐parasite arms race involves complex molecular crosstalk mediated by protein–protein interactions (PPIs). Bioinformatic analyses can be used to predict both host‐parasite PPIs and potential drug targets in parasite genomes, but high‐quality genomic data remain scarce for parasitic monogenean flatworms. Herein, an experimental lineage of *Gyrodactylus kobayashii* (Monopisthocotylea: Gyrodactylidae) is set up on goldfish hosts and used to conduct phased genome assembly using long‐range PacBio HiFi and Hi‐C technologies. In silico analyses of genomes of three *Gyrodactylus* species identified innexins as the most promising novel drug candidate genes. Drug screening and experimental verification singled out Imatinib as the most promising drug targeting innexins, with a high efficiency against *G. kobayashii* (100% mortality at 25 µM within 6 h in vitro) and low toxicity to the host. Prediction of PPIs in three *Gyrodactylus*‐host pairs revealed proteins associated with cAMP‐dependent signaling as key candidates, including the host's PRKACB and the parasite's PRKAR2A, RAP1A, ULK2, and Catenin Beta‐2. Two interacting G proteins are also identified: GNAO1 and GNB5. As the first high‐quality phased chromosome‐level genomic assembly for “monogeneans” and the first identification of PPIs in a fish‐parasite system, this study significantly advances the understanding of host‐parasite interactions at the genomic level.

## Graphical abstract

Genomic data offer a powerful tool for studying the molecular interactions between parasites and their hosts, but they remain scarce for parasitic monogenean flatworms. This study presents the first high‐quality phased genome assembly for monogeneans (Gyrodactylus kobayashii), and uses it to predict key interacting proteins between monogenean parasite ‐ fish host pairs, and identify novel drug targets.

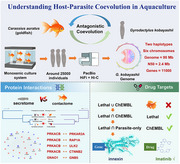

## 1 Introduction

Parasites have the ability to impact the physiology, behavior, reproductive success, and survival of their hosts. This generates strong evolutionary pressures on both sides, also known as the “antagonistic coevolution”: adaptations aimed at evading or resisting parasitic infections tend to increase the fitness of hosts, and counteradaptations aimed at evading or overcoming host defences tend to increase the fitness of parasites.^[^ ^1^, ^2^, ^3^ ^]^ Aside from being a topic of major importance for evolutionary biology, antagonistic coevolution also has important implications for medical and veterinary sciences.^[^ ^4^, ^5^ ^]^ A large part of this complex crosstalk between all symbiotic (comprising mutualism, commensalism, and parasitism) organisms comprises protein‐protein interactions (PPIs).^[^ ^6^, ^7^, ^8^ ^]^ This field remains poorly studied, partly because the experimental measurement of protein‐protein interactions suffers from a high false positive rate,^[^ ^9^ ^]^ and partly because it is difficult to study PPIs in non‐model animals.^[^ ^10^ ^]^ The onset of the genomic era facilitated in silico prediction of molecular interactions between symbiotic organisms and opened up multiple novel venues for identification of potential antiparasitic drug targets.^[^ ^11^ ^]^ As interacting proteins (“interologs”) in cellular networks, particularly those in network “hubs” (highly‐connected proteins), are generally highly conserved across different eukaryotic lineages,^[^ ^12^, ^13^ ^]^ this allows in silico prediction of interacting proteomes (interactomes) between species, provided that genomic sequences are available for them.^[^ ^8^, ^10^, ^14^ ^]^ This methodology has been used to study interactomes of humans and several of their major parasites,^[^ ^15^ ^]^ as well as several parasitic roundworms (Nematoda) and their non‐model mammal hosts,^[^ ^10^ ^]^ etc. However, little is known about the interactomes of non‐mammalian animals and their bilaterian parasites.

Among the parasitic animals, the obligate parasitic flatworms (Platyhelminthes: Neodermata) form by far the most speciose monophyletic parasitic clade.^[^ ^16^ ^]^ Unlike the well‐known parasites of mammals, cestodes and trematodes, the third neodermatan class, “monogenea” (paraphyletic, divided into classes Monopisthocotylea and Polyopisthocotylea^[^ ^17^, ^18^ ^]^), mostly comprises less well‐studied parasites of aquatic organisms. The genus *Gyrodactylus* (Monopisthocotylea: Gyrodactylidea: Gyrodactylidae) comprises 400 – 500 species of fish parasites.^[^ ^19^ ^]^ Due to their high speciosity, global distribution, the ability to infect a broad range of hosts, varied morphological structures, and varying degrees of host specificity (they encompass both highly host‐specific and generalist species), gyrodactylid parasites have been proposed as a good model for studying the coevolution and immune coadaptation between hosts and parasites.^[^ ^20^, ^21^ ^]^ Along with the evidence that fish can mount an effective immune response against gyrodactylids,^[^ ^22^, ^23^, ^24^ ^]^ this variability in host specificity suggests that molecular interactions play a key role in gyrodactylid‐host evolutionary dynamics.

With the development of aquaculture, gyrodactylid pathogens became a major veterinary problem, as they cause significant economic losses, far greater than any other “monogenean” genus.^[^ ^25^ ^]^ As a result, gyrodactylosis is among the 11 listed fish diseases by the World Organisation for Animal Health (OIE), highlighting the global significance of the threat that these parasites present to aquaculture. The primary method for treating monogenean infections in aquaculture comprises the use of traditional anthelmintic drugs (e.g., mebendazole, formalin, praziquantel, etc.), but there are major practical, environmental, and food safety problems associated with their large‐scale use. Aside from isolated reports of low efficiency and appearance of genotypes resistant to certain drugs, a major problem is low specificity, i.e., the fact that most of these drugs affect a wide range of nontarget species organisms, including the fish host, and even humans.^[^ ^26^, ^27^ ^]^ Thus, it is necessary to find viable alternatives to the existing drugs.^[^ ^26^, ^27^ ^]^ The onset of the genomics era facilitated the development of bioinformatic drug target discovery methods. In silico identification of potential drug candidates greatly reduces the scope of experimental targets, which speeds up the otherwise painstaking drug discovery process, while reducing its cost.^[^ ^28^ ^]^

Despite the diversity and veterinary importance of gyrodactylids, reference genomes are currently available for only two gyrodactylid species, *Gyrodactylus salaris* ^[^ ^29^ ^]^ and *Gyrodactylus bullatarudis*.^[^ ^30^ ^]^ Aside from these, only two more genomes are currently (December 2024) available for the entire class (or subclass, depending on the source) – Monopisthocotylea: *Benedenia humboldti* (GCA_016432935) and *Cichlidogyrus casuarinus* (GCA_045687925). This scarcity of genomic data is likely to be a consequence of sequencing difficulties associated with these small animals: a large number of specimens is commonly needed to produce a sufficient amount of DNA for standard genome sequencing methods.^[^ ^20^ ^]^ For example, the two previous gyrodactylid genomic studies used ≈15000 *G. salaris* and ≈3000 *G. bullatarudis* specimens to extract and pool the total DNA for NGS sequencing. However, the current long‐read HiFi sequencing technology demands a significantly larger genomic DNA input. Such a large number of specimens is commonly difficult or nearly impossible to sample in the wild, and they can exhibit highly pronounced genetic diversity. As a result of this scarcity of data and absence of a model species, little is known about molecular interactions between “monogenean” parasites and their hosts.

*Gyrodactylus kobayashii* is a highly host‐specific major parasite of the globally popular ornamental fish – goldfish (*Carassius auratus*).^[^ ^31^ ^]^ Due to the scattered and global nature of goldfish production (e.g., a lot of it taking place on small family farms),^[^ ^32^ ^]^ the estimates of global economic damages caused by *G. kobayashii* remain unknown, but isolated reports indicate that severe infections of *G. kobayashii* have a detrimental impact on the host and its aquaculture, whether by negatively affecting the ornamental value or by causing high mortality.^[^ ^33^ ^]^ For example, an outbreak of *G. kobayashii* in a single aquarium market in Henan Province, China, resulted in the goldfish mortality of nearly 80%.^[^ ^33^ ^]^ Further, its high host specificity implies a high level of molecular co‐adaptation with its host, and gene expression patterns in goldfish in response to *G. kobayashii* infection indicate that the parasite may have the ability to selectively inhibit certain elements of the host's immune responses.^[^ ^34^ ^]^ Combined, these observations make this host‐parasite system a good model to study the molecular basis of antagonistic coevolution between hosts and parasites. For this study, we set up a lab‐based host‐parasite system from a single *G. kobayashii* parasite (for the unique “Russian doll” reproduction mode of these viviparous protogynous hermaphrodites, see references [20, 35]), used it to sample over 20000 specimens, and sequenced their genomic DNA. As genomes are available for primary hosts of all three gyrodactylids mentioned herein: goldfish,^[^ ^36^ ^]^ *Poecilia reticulata* (host of *G. bullatarudis*),^[^ ^37^ ^]^ and *Salmo salar* (a major host of *G. salaris*),^[^ ^38^ ^]^ this allowed us to conduct a range of comparative genomic analyses using all three host‐parasite pairs. The primary objective was to in silico predict and characterize interactomes between the three gyrodactylids and their hosts, whereas the secondary objective was to in silico identify potential drug targets in the gyrodactylid genomes, predict existing drugs that may affect them, and test their efficiency. The results deepen our understanding of molecular mechanisms underpinning the antagonistic coevolution between hosts and parasites, and set the foundation for future functional studies aimed at the development of treatment and prevention options for flatworm parasites.

## 2 Results

### 2.1 The Chromosome‐Level Reference Genomic Assembly for *G. kobayashii*

**Figure 1:**
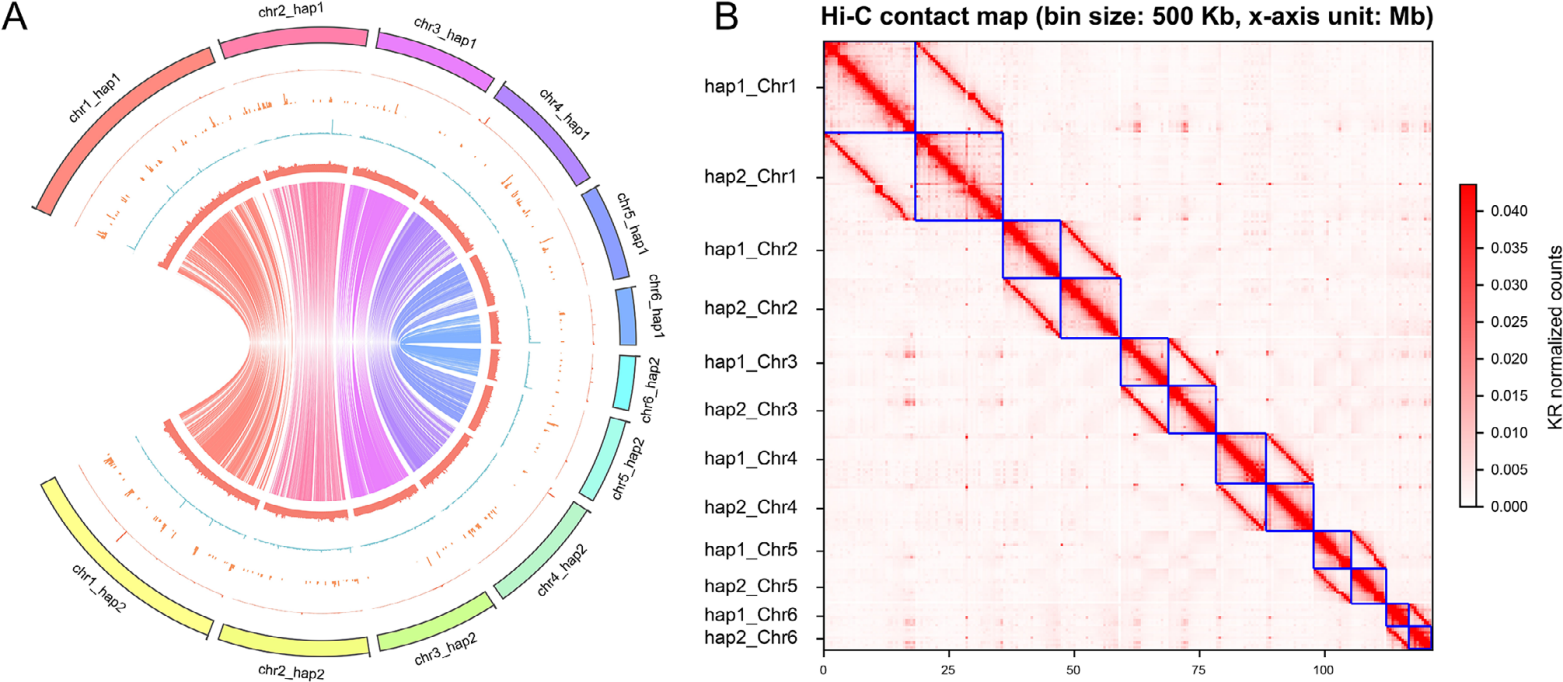
Features of the phased *G. kobayashii* genome assembly. A) The following information is presented from inside to outside: syntenous connections, GC content, gene density, repeat sequence density, HI‐C coverage depth, and karyotype. B) Hi‐C contact map of hap1 and hap2. Each block represents a Hi‐C contact between two genomic loci within each chromosome. The darker the colour of a block, the higher the contact intensity. Abbreviations: chr = chromosome; hap = haplotype.

Using a combination of HiFi sequencing and Hi‐C scaffolding, we produced a high‐quality, chromosome‐level reference genome of *G. kobayashii*. A total of 66.89 Gigabases (Gb) and 11.96 Gb of sequencing data were produced by HiFi and Hi‐C, respectively, and used to generate draft assemblies of two genomic haplotypes (hap1 and hap2). The two haplotypes exhibited 96 – 98% similarity in most overall parameters, aside from structural variations, where the differences were more strongly pronounced (60 – 90%) (**Table** **1**; Supplementary file 1: Tables S1 and S2, Supporting Information). The average proportion of SNPs in the raw sequences was 1.7/Kb. The total assembly sizes were around 90 Mb, but only ≈60 Mb (hap1 = 69.11%, hap2 = 64.07%) could be anchored to six chromosome‐level pseudomolecules for each haplotype (**Figure** **1**). In addition, in the absence of a parental genome assembly, chromosomes were randomly assigned to a haplotype. Repeats comprised 23 – 30% of the genome. The most abundant long terminal repeat (LTR) retrotransposon types present in the *G. kobayashii* genome were classified as “Gypsy” elements (≈3.5 – 4.0% of the genome), and the most abundant terminal inverted repeat (TIR) types were “Mutator” elements (≈15 – 21%) (Supplementary file 1: Tables S1 and S2, Supporting Information). A total of 10427/10943 genes were identified in nominal hap1/hap2, with the average CDS length near 2 kbp (Table 1). Among these genes, ≈60% were functionally annotated (Supplementary file 2: Datasets S1 and S2, Supporting Information). A total of 7497/8019 (71.90%/73.27%) hap1/hap2 (respectively) genes were homologous to the other two *Gyrodactylus* species, and 2930/2924 (28.10%/26.72%) genes (respectively) were unique to *G. kobayashii*. A total of 4397/4525 (42.2%/40.4%) of the hap1/hap2 genes had orthologues linked to one or more of the 358/360 KEGG biological pathways (Supplementary file 1: Figures S1–S2, Supporting Information).

**Table 1:** Overview of the genomic features and gene annotation statistics for the two *G. kobayashii* haplotypes (hap1 and 2) and the other two available gyrodactylid genomes: *G. bullatarudis* (GB) and *G. salaris* (GS). The data for the latter two species were extracted from the associated publication and annotation files for these two genomic assemblies. “Anchored” shows the statistics for contigs anchored to six chromosomes. BUSCO^g/p^ analyses were conducted using genomic assemblies/protein sequences respectively, and they are expressed as complete/fragment percentages (%).

Parameter	Hap1	Hap2	GB	GS
Genome size (Mb)	87.62	91.13	84.35	67.38
SNP/Kb	1.67	1.57	–	1.55
Anchored (Mb)	60.55	58.38	–	–
Anchored SNP/Kb	2.26	2.31	–	–
Number of scaffolds	190	167	4330	6069
Contig N50 size (Mb)	2.27	2.48	0.12	0.02
Scaffold N50 size (Mb)	9.46	9.30	0.31	–
GC content (%)	38.58%	38.48%	31.26%	33.85%
Repeat (%)	23.09%	29.42%	–	25.68%
Number of genes	10427	10943	10749	15436
Functionally annotated genes	6303	6511	7350	8278
Gene density (number/Mb)	119.02	120.09	126.00	228.80
Number of mRNAs	10427	10939	15787*	15414
Number of exons	77944	96178	85141	61601
Number of introns	67517	85249	69354	46187
Number of CDS	10427	10939	15787*	15414
mean gene length (bases)	4303	5061	4537	2662
mean mRNA length	4303	5058	5256	2662
mean exon length	269	251	347	228
mean intron length	353	368	773	586
mean CDS length	1845	2034	1490	909
mean mRNAs per gene	1	1	1	1
mean exons per mRNA	7	9	5	4
mean introns per mRNA	6	8	4	3
BUSCO^g^	58.7	58.6	58.6	55.6
BUSCO^p^	62.7	63.4	63.4	57.4

^*^Including alternative splicing.

### 2.2 Identification of Drug Targets and Experimental Confirmation

#### 2.2.1 Identification of Drug Targets in Gyrodactylid Genomes

In the first (least stringent) round of identification, the union of orthologs of ChEMBL database entries and “lethal” genes in the WormBase comprised 56 unique genes (according to KEGG annotation) in total: 35 ChEMBL hits and 24 WormBase hits (three genes overlapped between the two databases: 35 + 24‐3 = 56). In *G. bullatarudis*, 105 genes were identified: 66 ChEMBL hits and 46 WormBase hits (7 overlapped). In *G. salaris*, 103 genes were identified: 70 ChEMBL hits and 37 WormBase hits (4 overlaps). These 14 genes (3 + 7 + 4 = 14) that were found in both “lethal” and “CHEMBL” datasets (“Lethal∩ChEMBL”) comprised the second (more stringent) round of identification. Four genes in this subsection also exhibited the highest S_target_ scores (used to rank potential drug targets, for further explanations, see Methods) in the entire dataset: *rps‐7, rps‐2*, *nuo‐2*, and *kin‐20* (full gene names in **Table** **2**; we excluded genes that were likely to be misannotated from these analyses). Five genes passed the most stringent screening – ″lethal∩parasites‐only″ (lethal genes found only in parasites): *pmr‐1*, *iscu‐1*, and *F26F2.7*, and two innexin paralogues (Table 2). Among these, *pmr‐1* plays an essential role in embryogenesis in *Caenorhabditis elegans*,^[^ ^39^ ^]^ but it is a functionally highly conserved homolog of the SPCA1/ATP2C1 gene in vertebrates.^[^ ^40^ ^]^ Similarly, homologs of *iscu* exhibiting high sequence similarity to the human gene have been reported in fish.^[^ ^41^ ^]^ Finally, *F26F2* is a functionally poorly understood gene, but believed to be a homologue of vertebrate TAPT1. In all cases, these genes are likely to have orthologues in fish hosts and humans, so there may exist a risk of off‐target effects. On this basis, innexins were selected as the most promising candidates for further functional experiments.

**Table 2:** The identified candidates for drug targets in the three gyrodactylid genomes. The“Lethal ∩ parasites‐only” section of the table lists the intersection of genes associated with a lethal phenotype in WormBase and absent from fish host genomes; “Lethal ∩ ChEMBL” section comprises the intersection of genes identified as “lethal” and in the ChEMBL database; “Lethal ∪ ChEMBL” section comprises a union of remaining genes identified in these two databases with high S_target_ scores (≥4.0; full dataset in Supplementary file 2: Dataset S3, Supporting Information). The “Abbreviation” column shows gene name abbreviation, with a synonym given in brackets. The WBGene/ChEMBL/UniProt columns list gene identification numbers in WormBase, ChEMBL and UniProt databases respectively. “S_target_” scores could not be calculated for genes with no expression data (indicated by ″‐″); and ranges are given when multiple hits of the same gene produced different values. The “Species” column indicates the species in which the gene was identified as a drug candidate: GK is *G. kobayashii*, GB is *G. bullatarudis*, and GS is *G. salaris*.

Gene name	Abbreviation	WBGene	ChEMBL	UniProt	S_target_	Species
**“Lethal ∩ parasites‐only”**						
Innexin 13	*inx‐13 (opu‐13)*	00002135		A8XEA9	1.33‐2.12	GB+GK
Innexin 1	*inx‐1 (opu‐1*)	00002123		Q17394	1.14‐1.57	GB+GK+GS
Calcium‐transporting ATPase	*pmr‐1*	00004063			1.25	GB
Iron‐sulfur cluster assembly enzyme homolog	*iscu‐1*	00012885			2.75	GK
Protein TAPT1 homolog	*F26F2.7*	00009172			−	GS
**“Lethal ∩ ChEMBL”**						
Actin‐related protein 2/3 complex subunit 2	*arx‐4*	00021170	4295657	O15144	−	GK+GB
Eukaryotic translation initiation factor 5B	*iffb‐1*	0002185	4105852	O60841	3.04	GB+GS
Peptidase M24 domain‐containing protein; Xaa‐Pro dipeptidase	*K12C11.1*	00019673	4185	P12955	−	GB+GS
Casein kinase I isoform delta	*kin‐20* (*HRR25*)	00002203	3309063	P81123	4.68	GB+GK+GS
NADH dehydrogenase [ubiquinone] iron‐sulfur protein 3	*nuo‐2*	00020417	2363065	O75489	6.62	GB
Ribosomal protein small subunit 2	*rps‐2*	00004471	3987582	P15880	6.74	GB
Ribosomal protein Small subunit 7	*rps‐7*	00004476	3987582	P62081	7.99	GK+GB
Ribosomal protein S3A	*rps‐3A*	00004470	3987582	P61247	−	GS
H4 clustered histone 1	*his‐67* (*hist1h3d*)	00001941	5876	P62805	−	GS
Biopterin‐dependent aromatic amino acid hydroxylase family profile domain‐containing protein	*tph‐1*	00006600	3831287	Q8IWU9	3.01	GB
Mitogen‐activated protein kinase	*pmk‐2*	00004056	5789	Q00532	3.02	GK+GS
Serine/threonine‐protein kinase tousled‐like 1	*tlk‐1*	00006579	5404	Q86UE8	−	GK
Polo‐like kinase‐2	*plk‐2*	00004043	3024	P53350	−	GS
DNA polymerase alpha‐1	*pola‐1*	00012936	1828	P09884	−	GS
**“Lethal ∪ ChEMBL”**						
Stress‐induced phosphoprotein 1	*STIP1*		4523216	P31948	4.27	GB
Neuronal calcium sensor 2	*ncs‐2*	00003564			4.23	GB+GK+GS
GABA‐gated chloride channel, putative	*GABA‐Cl* (*lgc39*)	2364025	*8239847*	E0W492	1.55‐4.22	GB+GK
Butyrylcholinesterase	*bche*		4630814	P32750	2.1‐4.1	GK+GB+GS
Solute carrier family 18 member A3	*Slc18a3* (*unc‐17*)		2125	Q62666	4.03	GB
Acetyl‐CoA carboxylase beta	*ACACB (ACCase)*		2366573	Q3V4F7	4.35‐5.0	GB+GK+GS

#### 2.2.2 Innexins: Homology, Modeling, and Molecular Docking

Overall, innexins exhibited very low similarity to the host's genes; the highest similarity was to the host's pannexins (same gene superfamily), with no putative orthologues exhibiting sequence identity larger than 34%. They exhibited a high similarity to orthologues from other neodermatans: we identified a range of putative orthologues with >80% identity values (“monogenea” + Trematoda), and a few Cestoda orthologues in the 77–78% range.

As inx‐13 exhibited a higher level of conservedness across different species, we selected it for further analyses. Its 3D structure was predicted by homology modelling (Supplementary file 1: Figure S3, Supporting Information). A UniProt BLAST search identified the *Taenia asiatica* (Platyhelminthes: Cestoda) innexin protein as the best available template (78.52% sequence identity). The Global Model Quality Estimation (GMQE) score was 0.33. We used this model to conduct virtual screening of 2319 FDA‐approved drugs in the DrugBank, and the top 30 compounds with the lowest binding energy values (indicating best matches) were selected as potential candidates (**Table** **3**). From these, Atogepant, Conivaptan and Imatinib were prioritized for further evaluation based on the overlap of binding energy and availability, and subsequently tested for in vitro anthelmintic activity against *G. kobayashii*.

**Table 3:** The top 30 virtual drug screening hits. DB ID is DrugBank ID, and BA is Binding affinity in kcal/mol. Full details in Supplementary file 1: Table S3 (Supporting Information).

DB ID	BA	Name	DB ID	BA	Name
DB16098	−9.2	Atogepant	DB08881	−8.0	Vemurafenib
DB15328	−9.1	Ubrogepant	DB12457	−7.9	Rimegepant
DB00320	−9.0	Dihydroergotamine	DB11262	−7.9	Bisoctrizole
DB11986	−8.7	Entrectinib	DB14895	−7.9	Vibegron
DB00872	−8.5	Conivaptan	DB14703	−7.9	Dexamethasone metasulfobenzoate
DB15688	−8.5	Zavegepant	DB01117	−7.9	Atovaquone
DB14989	−8.4	Umbralisib	DB01199	−7.9	Tubocurarine
DB00966	−8.3	Telmisartan	DB04835	−7.9	Maraviroc
DB08827	−8.2	Lomitapide	DB00210	−7.9	Adapalene
DB09280	−8.1	Lumacaftor	DB00288	−7.8	Amcinonide
DB15011	−8.1	Avacopan	DB11262	−7.8	Bisoctrizole
DB06595	−8.1	Midostaurin	DB00619	−7.8	Imatinib
DB01126	−8.1	Dutasteride	DB11791	−7.8	Capmatinib
DB01336	−8.0	Metocurine	DB00549	−7.8	Zafirlukast
DB00762	−8.0	Irinotecan	DB00496	−7.8	Darifenacin

#### 2.2.3 In Vitro Anthelmintic Activity of Atogepant, Conivaptan, and Imatinib

**Figure 2:**
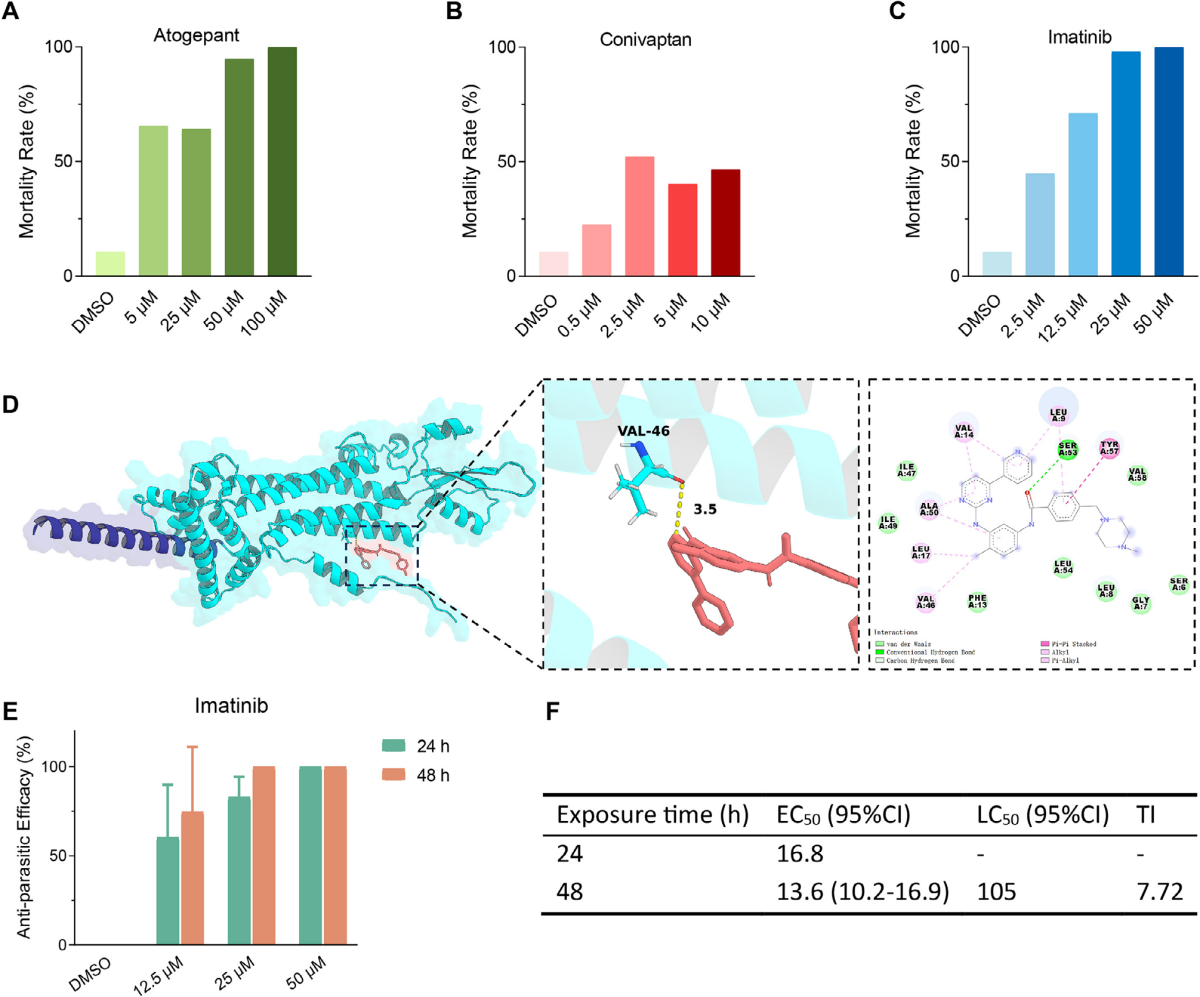
In vitro mortality rate (%) of *G. kobayashii* after a 6‐h exposure to varying concentrations of A) Atogepant, B) Conivaptan, and C) Imatinib. D) 3‐D INX‐13 model and its interaction patterns with Imatinib. E) In vivo mortality rates (%) of *G. kobayashii* after exposure to varying concentrations of Imatinib for 24 h and 48 h. F) Anthelmintic activity of imatinib against *G. kobayashii* and its acute toxicity to goldfish. Panels A, B, C, and E: DMSO was used as the control in all experiments, concentrations are shown on the x‐axis, and mortality (%) on the y‐axis. In vitro experiments (panels A‐C) were conducted with 24 replicates per group, and in vivo experiments (panels E and F) had 6 replicates. The goldfish toxicity assay comprised 10 replicates (panel F). In vivo experiments (panels E and F) comprised two time‐points: 24 and 48 h. Error bars show SD within a single experiment (panel E). In panel F, EC_50_ is the 50% effective concentration against parasites in µM, LC_50_ is the 50% lethal concentration for the host in µM, 95% CI is the 95% confidence interval, and TI is the therapeutic index (LC_50_/EC_50_).

The control group (0.02% DMSO) of *G. kobayashii* exhibited 10.7% baseline mortality after a 6‐h exposure period. Atogepant exhibited concentration‐dependent activity, achieving 94.9% and 100% mortality at 50 and 100 µM concentrations, respectively (**Figure** **2**A). Conivaptan had moderate effects that did not fully scale linearly with concentration: 22.5% (0.5 µM), 52.3% (2.5 µM), 40.3% (5 µM), and 46.7% (10 µM) mortality (Figure 2B). Imatinib caused 98.1% and 100% mortality at 25 and 50 µM concentrations, respectively (Figure 2C). On this basis, we selected imatinib for further experiments.

#### 2.2.4 Imatinib: Interaction Modeling, In Vivo Anthelmintic Activity, and Toxicity to Host

The interaction patterns between Imatinib and INX‐13 were visualized in both 2D and 3D representations (Figure 2D). Results indicate that Imatinib forms one hydrogen bond with the Val46 residue (3.5 Å). Additionally, multiple amino acids, including Ile47, Val58, Phe13, and Leu54, interact with INX‐13 through van der Waals forces and hydrophobic interactions.

In the in vivo experiment, Imatinib exhibited a time‐ and concentration‐dependent efficiency against *G. kobayashii* (Figure 2E). A 100% efficiency (complete parasite clearance) was observed at 50 µM following a 24 h exposure, and at 25 µM after a 48 h exposure. The 24‐h and 48‐h EC_50_ values were 16.8 and 13.6 µM, respectively (Figure 2F). Control groups exhibited no detectable anti‐parasitic activity throughout the observation period. Acute toxicity testing in goldfish revealed a favourable safety profile for Imatinib, with the 50% lethal concentration for the host (LC_50_) larger than 105 µM.

### 2.3 Protein Interactions Between Gyrodactylids and Their Hosts

**Figure 3:**
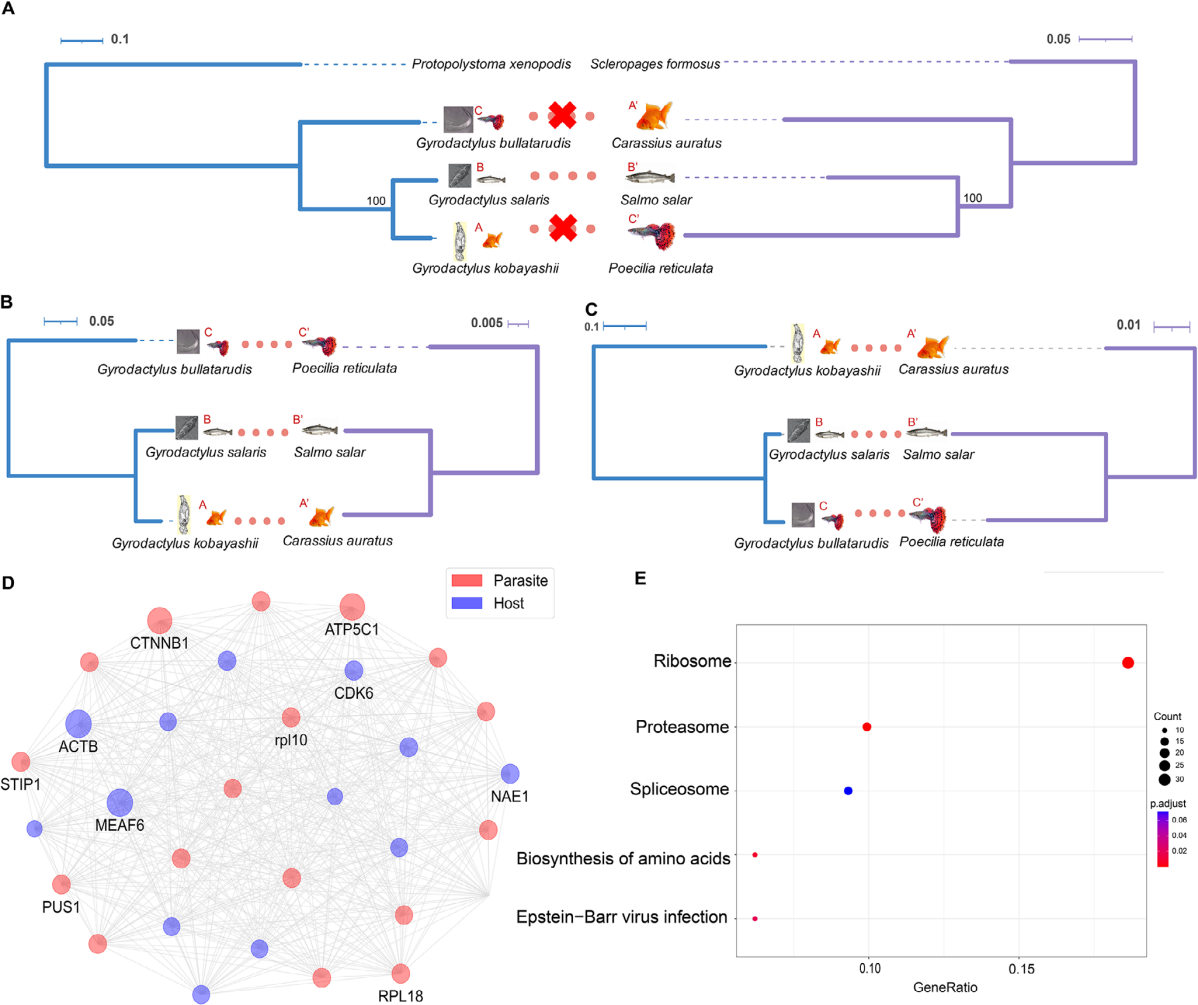
Cophylogeny screens, network analysis, and KEGG pathway enrichment. A) The non‐matching phylogenetic relationship between host and parasite species trees produced by the complete genomic datasets (1040 and 379 single‐copy orthologues, respectively, outgroups included). B) An example of a single‐gene host‐parasite cophylogeny matching the parasite species tree (AB_C, See “5. Experimental Section” section for the definition of A, B and C). C) Host‐parasite cophylogeny example matching the host species tree (BC_A). In panels A, B and C, mismatches between parasites and hosts are indicated by crosses, and matches by dots. Branch length bars are shown next to the phylograms. D) PPI network analysis showing the top 10% of proteins identified in the interactome (Contactome+Secretome) between *G. kobayashii* and *C. auratus* ranked by the betweenness centrality after applying the phylogenetic congruence filter. The node size is positively correlated to the betweenness centrality value, and node colour corresponds to the host/parasite side of the PPI (see legend in the figure). Protein names are shown for the top‐ten ranked nodes. E) Significantly enriched KEGG pathways among the identified protein‐protein interaction (PPI) candidates between gyrodactylids and their hosts in hap1. The query dataset comprised only PPI candidates that passed the phylogenetic congruence test, and the background comprised merged secretome and contactome datasets (duplicates removed). GeneRatio (x‐axis) represents the ratio of the number of genes involved in the functional annotation and the total number of genes tested, where the first number is roughly represented by the size of the node. Only pathways exhibiting adjusted *p*‐values <0.1 are shown. The size and the color of the node correspond to the number of genes and adjusted *p*‐value, respectively (the legend is shown to the right of the graph).

We constructed protein‐protein interaction (PPI) networks between hosts and gyrodactylids using their genome‐wide filtered proteomes (only hap1 was used for *G. kobayashii*). As host and parasite pairs did not exhibit congruent phylogenies at the phylogenomic (species) level (**Figure** **3**A; Supplementary file 1: Figure S4, Supporting Information), we screened for putatively co‐evolving pairs of host‐parasite genes by identifying those that exhibited matching phylogenies (Figure 3B,C), as it may indicate coevolutionary pressures acting on pairs of interacting proteins. Among the 1869 PPI pairs identified in the secretome dataset, 669 (35.79%) passed the cophylogeny screen; among the 31 PPIs in the contactome dataset, 8 (22.8%) passed the cophylogeny screen (**Table** **4**; Supplementary file 2: Datasets S4–S9, Supporting Information). Among these, PPI pairs that conformed to the parasite species topology were more prevalent in both datasets. Among the parasite/host PPI genes in the merged secretome+contactome dataset, 93.8/38.3% conformed to the parasite/host species topology respectively; a significantly lower proportion than among all 1040/379 single‐copy orthologues used to infer the parasite/host species trees: 99.1/51.2% respectively (both p<0.001, z‐values = 5.7/3.4 respectively; Table S4, Supporting Information). To further confirm the difference in the phylogenetic signal, we concatenated the genes identified in parasite and host PPI datasets into two supermatrices, inferred phylogenies, and found that both datasets produced the parasite species topology (Supplementary file 1: Figure S5, Supporting Information).

**Table 4:** Topology tests. “PPIs cophylogeny” rows show the total number of PPI protein pairs exhibiting matching topologies, and the proportions of matching topologies corresponding to three topological types. “PPI Parasite/Host genes” rows show the total number of genes among the respective secretome (1869), contactome (31), and secretome+contactome (1900) PPI datasets, and the proportion of topological types among these genes; “Total Parasite/Host SCO” rows in the “Complete genome” section of the table show the prevalence of topological types among all single‐copy orthologues identified across respective complete genomic datasets used for phylogenetic analyses. Among the columns, “Total” shows the total number of PPI pairs or genes in the dataset; “AB_C%” shows the percentage of PPIs/genes corresponding to the parasite species topology (A and B species are the closest), “BC_A%” to the host species topology, and “AC_B%” to the “random” topology (See Figure 3). See “5. Experimental Section” section for the definition of A, B and C.

Dataset	Total	AB_C%	BC_A%	AC_B%
**Secretome**				
PPIs cophylogeny	669	97.9	1.5	0.6
PPI Parasite genes	280	94.64	2.86	2.14
PPI Host genes	298	46.31	35.91	16.44
**Contactome**				
PPIs cophylogeny	8	62.5	25	12.5
PPI Parasite genes	10	70	10	20
PPI Host genes	31	35.48	61.29	3.23
**Merged Secretome and Contactome**				
PPI Parasite genes	290	93.79	3.10	2.76
PPI Host genes	329	45.29	38.3	15.2
**Complete genome**				
Total Parasite SCO	1040	99.13	0.48	0.39
Total Host SCO	379	42.74	51.19	6.07

The betweenness centrality analysis results indicated that Catenin Beta 1 (CTNNB1) was among the top ten hits, both before and after applying the phylogenetic congruence filter (Figure 3D; Supplementary file 1: Figure S6, Supporting Information). The 280 parasite genes found in the secretome dataset that passed the phylogeny screen (Table 4) were most significantly functionally enriched in the Ribosome and Proteasome pathways (Figure 3E) against the background gene set comprising the merged contactome and secretome datasets before the phylogeny screen (290 + 329 = 619, Table 4). To attempt to further reduce the number of false positives (i.e., within‐species interactions identified as host‐parasite interactions), we identified pairs of PPI proteins that appeared in both fish contactome and parasite secretome datasets (after the phylogeny screen). This yielded a list of five PPIs in the secretome result, and none in the fish contactome result (**Table** **5**). Among them, four pairs comprised the host protein cAMP‐dependent protein kinase (PRKACBB) interacting with four different parasite proteins: ULK2, RAP1A, PRKAR2A (also a cAMP‐dependent protein kinase), and a paralogue of CTNNB1 – Catenin Beta 2 (CTNNB2). The fifth PPI pair comprised two G protein family members: host GNAO1A and parasite GNB5.

**Table 5:** The screened candidates for protein‐protein interactions (PPI) between fish hosts and *Gyrodactylus* parasites. All candidates passed the phylogenetic congruence test and appeared in both the parasite secretome and the fish contactome lists. For each interacting protein/gene, the preferred STRING database abbreviation and the full name are shown.

Host		Parasite	
Abbreviation	full name	abbreviation	full name
PRKACBB	cAMP‐dependent, catalytic, beta b (protein kinase superfamily)	PRKAR2A	cAMP‐dependent, regulatory, type II, alpha A (protein kinase)
PRKACBB	*Ditto*	RAP1A	RAP1A (RAS oncogene family a)
PRKACBB	*Ditto*	ULK2	Unc‐51‐like autophagy‐activating kinase 2
PRKACBB	*ditto*	CTNNB2	Catenin, beta 2
GNAO1A	Guanine nucleotide‐binding protein “G protein” (alpha‐activating activity polypeptide O, a)	GNB5	Guanine nucleotide‐binding protein subunit beta‐5a

## 3 Discussion

### 3.1 Setting Up an Experimental Lineage of G. kobayashii and Phased Genomic Assembly

Due to the small body size of *G. kobayashii* (0.3 – 1 mm), individual parasites contain only ≈ 1000 cells, which results in very low DNA yields per specimen,^[^ ^20^ ^]^ insufficient for high‐quality complete genome sequencing using standard methods. While this problem can be overcome using whole‐genome amplification techniques, they are prone to introducing a range of amplification artifacts,^[^ ^29^, ^42^ ^]^ so we opted to extract DNA from a large number of specimens. The same approach was employed by the previous two studies that sequenced gyrodactylid genomes (*G. salaris* and *bullatarudis*). It should be noted that they employed older NGS techniques,^[^ ^29^, ^30^ ^]^ whereas we relied on the long‐range Pac‐Bio sequencing and Hi‐C phased genome assembly approaches, which require an even larger amount of DNA. Such a large number of specimens is difficult to sample in the wild, and they can exhibit highly pronounced sequence diversity. To address these two problems, we set up an experimental lineage from a single *G. kobayashii* parasite collected from a goldfish specimen, which may be relevant for the overall scientific progress on “monogeneans”. This allowed us to collect a large number of specimens, which in turn enabled us to produce the first chromosome‐level phased genomic assembly for “monogeneans”.

There are several sources of noise in our data. As mentioned before, in the absence of the parental genotype, chromosomes were randomly assigned to haplotypes. Further, the DNA was extracted from more than ten thousand specimens, which is likely to result in sequence variability. Although we set up this lineage in a way to minimize genetic variability, the genetic diversity of the raw dataset closely corresponded to the one reported for *G. salaris*.^[^ ^29^ ^]^ An additional source of noise is the fact that gyrodactylids commonly reproduce both mitotically and meiotically,^[^ ^35^ ^]^ so we cannot exclude the issue of recombination affecting our haplotype assembly results. Finally, we managed to anchor only near 70% of contigs to the six identified chromosomes and found non‐negligible levels of structural variation between the two haplotypes, which suggests the existence of chromosomal plasticity among individual parasites or pollution from the host genome. These issues highlight the need for additional high‐quality monogenean genomic assemblies, necessary to parse noise from the genomic plasticity signal.

### 3.2 Comparative Genomics of Gyrodactylids

The assembly size and number of annotated genes of the two genomic haplotypes of *G. kobayashii* were more similar to *G. bullatarudis* than to *G. salaris*.^[^ ^29^, ^30^ ^]^ The number of genes was comparable to other parasitic flatworms.^[^ ^11^, ^43^ ^]^ The number of scaffolds was much smaller in *G. kobayashii* than in the other two gyrodactylid genomes, which is a direct consequence of the reliance on long‐range PacBio HiFi and Hi‐C technologies in this study, but BUSCO values were similar to *G. bullatarudis*. We suspect that the high number of nominally “unique” genes identified in *G. kobayashii* is more likely to be indicative of the scarcity of data and annotation difficulties in these species than an actual evolutionary feature, but further studies and improved gene annotation tools are needed to thoroughly assess this issue.

**Figure 4:**
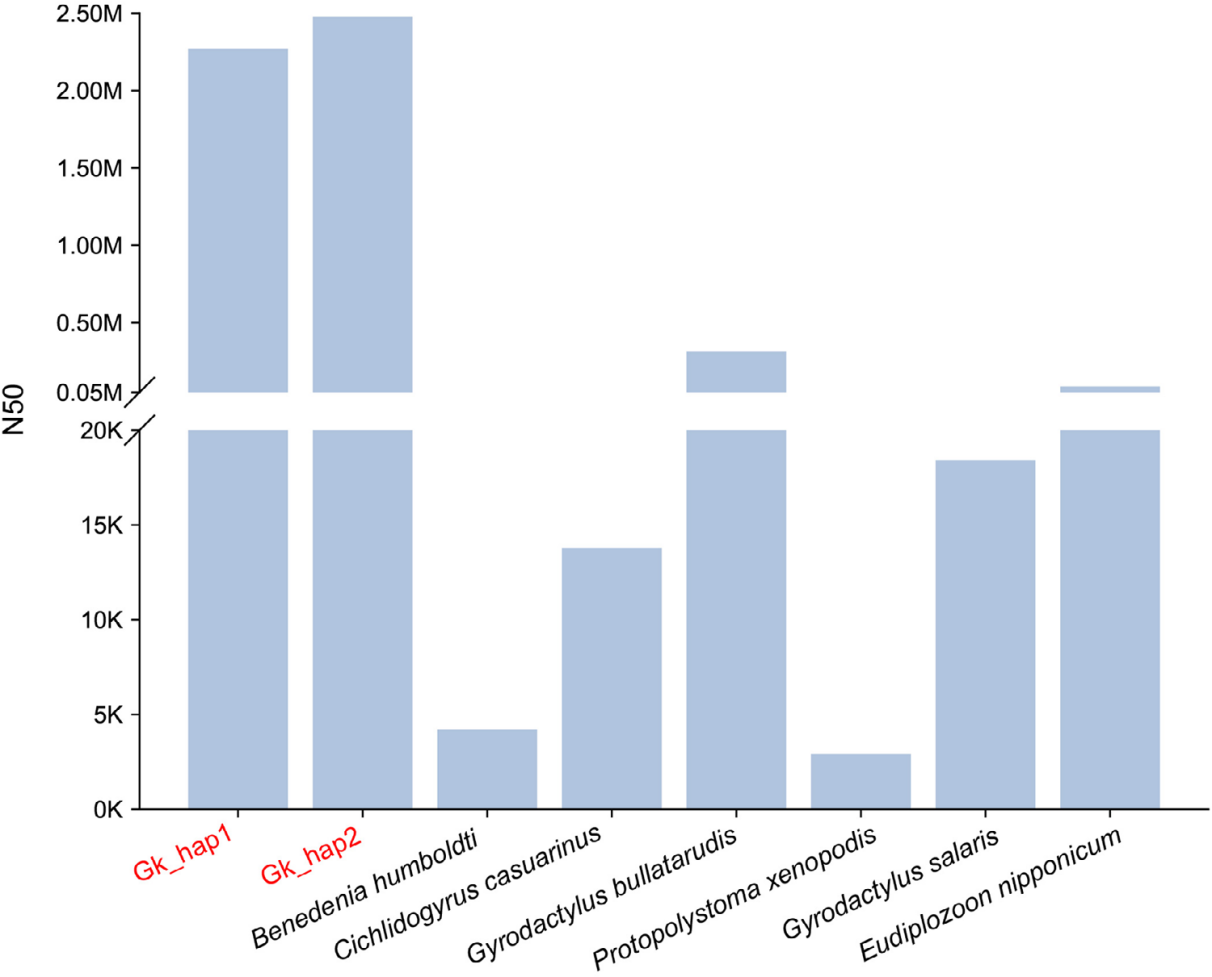
Contig N50 values for the six available “monogenean” genomes and the two *G. kobayashii* haplotypes (Gk_hap1/2). The y‐axis is cropped, with the lower part exhibiting N50 values in Kilobases (K), and the upper part in Megabases (M).

Notably, the contig N50 value of our *G. kobayashii* assembly was between eight times and over ten thousand times higher than in the other six available monogenean genomes (**Figure** **4**), and the mean CDS length was 350–950 bp larger than in the other two gyrodactylids. This suggests that improved structural quality of the assembly also resulted in improved annotation (i.e., fewer fragmented genes). In comparison to the much better studied cestodes and trematodes, the two classes comprising “monogeneans” (Monopisthocotylea and Polyopisthocotylea) are markedly understudied, with only six genome assemblies currently available (Dec. 2024), all of which were produced using old NGS sequencing methods. This highlights the importance of the availability of a high‐quality genomic assembly.

### 3.3 Identification of Potential Drug Targets in the Genome of G. kobayashii

As innexins are an invertebrate‐specific gene family,^[^ ^44^ ^]^ this potentially makes them a promising drug target, as it strongly reduces the likelihood of toxicity to hosts and humans. Innexins play crucial roles in the molecular channel forming, and as such, partake in a wide range of physiological processes, comprising cellular communication, embryo development, behavior, immune responses, and apoptosis.^[^ ^44^, ^45^ ^]^ As regards innexin 13 (*inx‐13*; synonyms: let‐585, opu‐13, CELE_Y8G1A.2), it was identified as a gene causing a lethal phenotype in the larvae of *C. elegans*.^[^ ^46^, ^47^ ^]^ WormBase indicates that the gene was identified as a lethal gene in adult *C. elegans* as well, or that it causes sterility, but we failed to confirm this in related studies.^[^ ^48^, ^49^, ^50^, ^51^ ^]^ As regards the *inx‐1* (synonyms: opu‐1, pcr55, CELE_C16E9.4), it was identified as a gene whose mutations cause maternal‐effect embryonic lethality.^[^ ^52^ ^]^ Importantly, innexins may also be a suitable target in other parasitic flatworms of major veterinary and medicinal importance. Indeed, two innexins (inx‐3 and inx‐4) were proposed as putative drug targets in the parasitic nematode causing elephantiasis in humans: *Brugia malayi* (Nematoda).^[^ ^53^ ^]^ Further, schistosomiasis is a human helminth disease with major medical importance, infections of which are commonly treated with praziquantel, an old drug that is ineffective against juvenile worms.^[^ ^54^ ^]^ On this basis, it was proposed that an anthelmintic drug designed to supplement praziquantel, but targeting early development stages, would both increase efficacy and reduce the risk for resistance to this key anthelmintic drug.^[^ ^54^ ^]^ This further highlights the high potential of innexins as drug targets for parasitic flatworms.

The remaining genes identified in both “lethal” and “ChEMBL” databases also exhibited (some of) the highest S_target_ scores in the dataset: *rps‐7, rps‐2*, *nuo‐2*, and *kin‐20*. The two *rps* paralogues both belong to the family of ribosomal proteins, crucial for ribosome assembly and function, but also implicated in tumorigenesis, immune signaling, and development. As such, they have been implicated in multiple diseases in humans.^[^ ^55^, ^56^ ^]^ NADH dehydrogenase [ubiquinone] iron‐sulfur protein 3 (*nuo‐2*) is associated with mitochondrial metabolism, i.e., energy production, and it has received some scientific attention for its association with life span in *C. elegans*.^[^ ^57^ ^]^ Casein kinase 1δ (kin‐20) is a key enzyme for architectural stability and maturation of the nervous system.^[^ ^58^ ^]^ All these genes are functionally highly conserved across the animal kingdom, so their direct targeting would first require careful assessment of toxicity to the host and humans.

There were several sources of noise in these analyses. First, we argue that a deeper understanding of the functional repertoire of monogenean genomes would be likely to produce a much wider range of potential drug targets, as a large proportion of gene families in genomes of parasitic helminths remains functionally unannotated.^[^ ^11^ ^]^ This scarcity of data for parasitic flatworms also forced us to rely on the distantly related model species, a free‐living nematode *C. elegans*, to identify putatively lethal genes, which may have reduced the precision of our methodology.

Due to the lack of genomic data, previous research on drug targets and anthelmintic screening for *G. kobayashii* primarily relied on identifying potential drug target genes reported in studies of other helminths. This approach often led to failures or weak target specificity in subsequent molecular docking and anthelmintic assays.^[^ ^59^ ^]^ The high‐quality genomic assembly of *G. kobayashii* presented in this study provides a crucial resource for this work, significantly enhancing the accuracy of drug target identification and increasing the success rate of effective drug screening. As the genomic repertoire of monogenean parasites remains very poorly understood, this genome will also serve as the foundation for future drug identification studies in monogenean parasites.

### 3.4 Drug Screening for Innexins Using Molecular Docking and Functional Confirmation

Based on the above results, we selected innexins to conduct molecular docking and screen for potential innexin‐targeting drugs. This is an emerging approach in parasitology that enables efficient preliminary screening of available drugs, but its predictions require experimental validation.^[^ ^60^ ^]^ Experimental confirmation of screened drug candidates showed that Atogepant (100 µM) and Imatinib (25 µM) induced 100% mortality within 6 h in vitro, whereas Conivaptan did not exhibit a concentration‐dependent efficiency. Due to the high cost of Atogepant, it may not be suitable for large‐scale application in aquaculture, so we chose Imatinib for subsequent in vivo verification.

Imatinib is a drug used for several types of tumor in humans, which share dysregulated activity of an imatinib‐sensitive kinase as the underlying pathogenic feature.^[^ ^61^ ^]^ It has also shown efficacy against *Echinococcus* stem cells, metacestode vesicles, and protoscoleces.^[^ ^62^ ^]^ Herein, we found that it was also highly efficient against *G. kobayashii* in vivo, while exhibiting low toxicity to the hosts. A downside is that its EC_50_ (6.71–8.29 mg L^−1^) was higher than that of several other drugs previously applied in the treatment of gyrodactylids, such as Isoimperatorin (EC_50_ = 0.53 mg L^−1^), Plumbagin (EC_50_ = 0.09 mg L^−1^), and the commonly used anthelmintic mebendazole (EC_50_ = 0.023 mg L^−1^).^[^ ^59^, ^63^, ^64^ ^]^ In addition, we cannot be certain that the effects were achieved via the disruption of inx‐13 functionality, or via off‐target effects on some other key molecules.^[^ ^61^ ^]^ Regardless, the high therapeutic index (TI = 7.72) observed in our experiments, low cost, and broad commercial availability of Imatinib make this drug an attractive option for anthelmintic applications in fish.

### 3.5 Key Interacting Proteins Between Gyrodactylids and Their Hosts

Among the most remarkable evolved mechanisms within the host‐pathogen dynamics is a whole repertoire of finely tuned immunomodulatory molecules that allow pathogens to manipulate the host's immune response in their favour.^[^ ^65^, ^66^ ^]^ As these interactions underpin most key ecological traits in host‐parasite interactions, such as the prevalence, virulence, host‐specificity, etc., this may represent another venue for identification of potential drug candidates in parasites. In order to improve the scientific understanding of these mechanisms, we constructed protein interaction networks between three host‐gyrodactylid pairs. Due to the limitations intrinsic to the methodology employed, we suspected that the full results comprised both intra‐ and inter‐species PPI candidates. This problem has been mentioned before,^[^ ^15^ ^]^ but we are of the opinion that the difficulty in parsing false positives from true positives was explained too vaguely, and the potential severity of the problem was insufficiently highlighted in that study. To address this issue and reduce the proportion of false positives, we first followed the example of Hu et al.^[^ ^10^ ^]^ and narrowed down the selection to only genes exhibiting phylogenetic congruence, under the hypothesis that it signifies coevolution between hosts and parasites.^[^ ^67^ ^]^ We must be wary of false positives here, because only about half of the host gene trees conformed to the host topology in the full genomic dataset. However, we did find a significant increase in the proportion of genes conforming to the “wrong” topology in both host and parasite PPI datasets (i.e., parasite genes conforming to host topology and vice versa), which is indicative of the presence of coevolutionary forces in the PPI dataset. In more detail, we found that putative gene interactions affected the topologies in the predicted manner: parasite PPI gene trees conformed to the “host species topology” more often than in the complete dataset (3.10% vs 0.48% respectively; Table 4: column “BC_A%”), and host PPI gene trees conformed to the “parasite species topology” more often than in the complete dataset (45.3% vs 42.7% respectively; Table 4: column “AB_C%”). However, the proportion of “random” (AC_B) topologies was increased among both parasite and host PPI genes compared to the complete gene set. This suggests that secretome and contactome evolve under unique evolutionary pressures, not directly related to the interaction between this particular host‐parasite pair. Thus, to attempt to further screen false‐positives, we designed an additional filter, which allowed us to focus narrowly on the pairs of genes that were identified in both the fish contactome (the “fish” PPI member) and parasite secretome (the “parasite” PPI member). While this does not fully resolve the problem of false positives, we hope that our improved methodology has helped us reduce the noise from false positives in PPI identification in comparison to previous studies. Another limitation that should be mentioned is that the methodology may not capture all host/parasite molecules that come into contact.

Among the five identified pairs of proteins, four of them comprised a catalytic cAMP‐dependent protein kinase named PRKACB (protein kinase, cAMP‐dependent, catalytic, beta b) on the host side of the PPI. This family of proteins is a major component of the cAMP‐dependent pathway, a G protein‐coupled receptor‐triggered signaling cascade involved in cell communication. As regards the fifth pair of proteins, it consisted of two guanine nucleotide‐binding proteins (GNAO1 and GNB5), also known as G proteins. Therefore, all five identified candidate protein pairs comprised proteins involved in the G protein/cAMP signaling pathways. In addition, in one case, PRKACB was paired with another cAMP‐dependent protein kinase, PRKAR2A.

The external surface of the tegument, the outer body covering of parasitic flatworms (Neodermata), transduces environmental signals. It is rich in secretory proteins, comprising G protein‐coupled receptors (GPCRs).^[^ ^68^, ^69^ ^]^ GPCRs are a large superfamily of transmembrane proteins of eukaryotes, responsible for detecting many types of extracellular signals and transducing them to the heterotrimeric G proteins, a family of proteins highly conserved across eukaryotes, and involved in downstream signal transduction.^[^ ^68^, ^70^ ^]^ Ligands that bind to GPCRs comprise neurotransmitters, odorants, pheromones, and hormones.^[^ ^68^, ^71^ ^]^ GPCR activation triggers a signaling cascade involved in cell communication known as the cAMP‐dependent pathway, highly conserved across the animal kingdom, with wide regulatory roles, comprising a central role in signal transduction in response to inner and outer stimuli.^[^ ^72^ ^]^ As such, G proteins have wide‐ranging regulatory functions, including signal transduction, metabolic transport, cell movement, chemosensory perception, protein synthesis, and growth and differentiation, in most eukaryotes.^[^ ^73^ ^]^ This central role of G proteins, GPCRs, and cAMP‐dependent protein kinases in our results corresponds to findings from previous studies, where these classes of molecules have been implicated in human host invasion by the malarial *Plasmodium falciparum* parasite,^[^ ^74^, ^75^ ^]^ in host detection of free‐living parasitic life stages,^[^ ^76^ ^]^ as well as for drug targets for trematodes (schistosomes).^[^ ^77^ ^]^ Further, due to their role as chemosensory receptors in most animals, GPCRs have been proposed as a promising research focus for understanding how free‐living parasitic life stages find hosts.^[^ ^76^ ^]^ They also play key roles in single‐cellular parasites; for example, heterotrimeric G protein signaling likely regulates amoebic motility, as well as attachment to and killing of host cells.^[^ ^78^ ^]^ In addition, GPCRs, in combination with ion channels, protein kinases, and nuclear hormone receptors, account for almost half of all protein drug targets in humans.^[^ ^79^ ^]^ GPCR‐based drugs targeting parasitic flatworms often regulate cAMP levels, thus interfering with transitions between different life stages.^[^ ^80^ ^]^ Finally, there are indications that the G‐protein family is highly significantly expanded in helminth parasites in general and that it comprises multiple helminth‐specific gene families.^[^ ^11^ ^]^ There is evidence that gyrodactylids are not an exception in this aspect, as the GPCR pathway comprised a disproportionate number of duplicated genes in *G. bullatarudis*.^[^ ^30^ ^]^ Combined, these findings strongly indicate that this pathway may play a key role in the interaction between gyrodactylid parasites and their fish hosts.

As regards the specific candidate proteins/genes, PRKACB emerged as the key protein, as it was identified as the “host” side of the PPI in four PPI pairs. PRKACB is a catalytic subunit of cAMP‐dependent protein kinase A (PKA), which mediates signaling through cAMP.^[^ ^81^ ^]^ PRKACB has been associated with apoptosis and MAPK signaling pathways in fish.^[^ ^82^ ^]^ The manipulation of apoptotic pathways by parasites is a well‐characterized mechanism that they employ to evade the host's immune responses, whether by preventing apoptosis in host cells inhabited by parasites or by promoting apoptosis in host immune cells programmed to attack them.^[^ ^83^, ^84^, ^85^ ^]^ Similarly, Mitogen‐Activated Protein Kinases (MAPK) signaling has multifaceted roles in innate immune responses. The pathway is activated by a range of pattern recognition receptors (such as Toll‐like receptors – TLRs), and its downstream effects may be both anti‐inflammatory and pro‐inflammatory (e.g., activation of cytokines and chemokines).^[^ ^86^ ^]^ It is known from previous studies that *G. kobayashii* infections result in a significant regulation of both pro‐ and anti‐inflammatory cytokines in goldfish, but the regulatory effects remain contradictory.^[^ ^87^, ^88^ ^]^ A possible explanation may be that elevated expression levels of pro‐inflammatory genes were only detected at early stages of infection.^[^ ^34^ ^]^ Overall, this indirectly lends support to the hypothesis emerging from our results that the host's PRKACB may play an important, and possibly even central, role in goldfish‐*G. kobayashii* molecular interaction mechanism. The four parasite proteins that PRKACB was paired with comprised: PRKAR2A, RAP1A, ULK2 and CTNNB2.

PRKAR2A is one of the four main regulatory subunits of PKA,^[^ ^81^ ^]^ which explains its interaction with PRKACB. Our results indicate the existence of multiple paralogues of this gene in *G. kobayashii*, so we cannot ascertain which of the regulatory subunits this is with full confidence. Just like PRKACB, PRKAR2A is highly conserved across a broad range of life forms, and it is involved in multiple regulatory functions, including the regulation of inflammation.^[^ ^89^, ^90^ ^]^ For example, PRKAR2A can directly bind to the interferon γR2, thus suppressing the JAK2/STAT1 signaling and alleviating inflammation.^[^ ^91^ ^]^ Therefore, there are multiple ways in which this PPI pair may be employed by gyrodactylids to interfere with the host's immune responses.

RAP1 (Ras‐related protein Rap‐1A is a member of the Ras family of small guanine triphosphatases (GTPases), highly conserved across the animal kingdom.^[^ ^92^ ^]^ Ras family has exceptionally wide‐ranging metabolic roles, including the extracellular signal recognition, the control of cell‐cell and cell‐matrix interactions, MAPK activity, and immune responses via its positive influence on T cells.^[^ ^92^, ^93^ ^]^ For example, RAP1 plays a key role in host defense system evasion in *Trypanosoma brucei*.^[^ ^92^ ^]^ In mammals, RAP1 has two paralogues, RAP1A and RAP1B, both of which were associated with late endocytic/phagocytic processes.^[^ ^94^ ^]^ Both RAP1A and B proteins are phosphorylated by the PKA,^[^ ^95^ ^]^ which explains the inferred interaction with PRKACB. RAP1A was recognized as a drug target for an endocellular parasite *Leishmania*,^[^ ^96^ ^]^ and it is a substrate of geranylgeranyltransferase (GGTase‐I), which was also proposed as a target for countering parasitic infections.^[^ ^97^ ^]^ Its paralogue, RAP1B, mediates cAMP‐dependent invasion by *Trypanosoma cruzi* in host cells.^[^ ^98^ ^]^

ULK2 (Unc‐51‐like autophagy‐activating kinase 2) is involved in the positive regulation of autophagy.^[^ ^99^, ^100^ ^]^ The mechanism of autophagy is integrated with pattern recognition receptors and cytokine signaling, and employed as an innate immunity strategy to eliminate intracellular pathogens. In response, microparasites developed strategies to impair the autophagic machinery in phagocytes.^[^ ^101^, ^102^ ^]^ The role of autophagy in response to helminth parasites is less well‐understood, but a similar gene, ULK2‐like, was upregulated in goldfish in response to a *G. kobayashii* infection.^[^ ^88^ ^]^ In addition, cAMP signaling, specifically the Ras/PKA signaling pathway, plays a role in the negative regulation (inhibition) of autophagy,^[^ ^103^ ^]^ which may explain the interaction of ULK2 and PRKACB. However, as this interaction was inferred from their homologues in yeast,^[^ ^104^ ^]^ its specifics remain unknown.

Finally, PRKACB was also paired with parasite's β‐Catenin 2 (CTNNB2). The canonical role of this gene comprises the regulation of left‐right (LR) asymmetric development via the β‐Catenin‐mediated canonical Wnt signaling,^[^ ^105^ ^]^ but later it was discovered that a protozoan parasite *Toxoplasma gondii* relies on the conserved GSK3/β‐catenin axis to reshape the host cell interactome and selectively reprogram immune gene expression.^[^ ^106^ ^]^ Notably, its paralogue, CTNNB1, was among the top ten PPI candidates with the highest betweenness centrality (also identified as the “parasite” side of the PPI). It remains to be experimentally tested whether helminth parasites may also rely on this mechanism to interact with the host's immune reaction.

As regards the pair of interacting G proteins, the host side of the PPI, GNAO1, has been identified as a key component in the activation of TLR signaling, a highly conserved key mechanism in the host defense against pathogens, with a particularly prominent role in fish.^[^ ^107^, ^108^, ^109^, ^110^ ^]^ There is also evidence that this pathway plays a role in the goldfish response to *G. kobayashii* infection.^[^ ^88^ ^]^ As GNB5 is involved in the termination of signaling initiated by the G protein‐coupled receptors,^[^ ^111^ ^]^ we hypothesize that it may be employed by gyrodactylids to interfere with the host's TLR signaling. STRING database indicates that its interaction with GNB5 was inferred on the basis of their co‐expression in a range of organisms, but mostly in association with photoreceptors in retina, e.g.,^[^ ^112^ ^]^ so the specific mode of action remains unknown.

## 4 Conclusion

Innexins emerged as the most promising drug targets in the genomes of parasitic gyrodactylids, and Imatinib was identified as the most promising drug targeting innexins. Imatinib exhibited a high efficiency against *G. kobayashii* and low toxicity to the host. As regards the remaining candidate genes, the off‐target effects of any drug targeting them, i.e., toxicity to the host and humans, should first be carefully assessed. In the PPI results, GPCR / cAMP signaling pathways were disproportionately prevalent among the top hits. Given the known functions of this pathway, the identified proteins may serve as molecular targets employed by *G. kobayashii* to evade the host's immune system. As is common in pioneering forays, this study opens far more questions than it manages to close. Therefore, we hope that setting up an experimental *G. kobayashii* lineage and producing a high‐quality genomic assembly will be the crucial first two steps on a long journey to explore this vast research area.

## 5 Experimental Section

### Parasite Culturing and Collection

In order to obtain a sufficient number of *G. kobayashii* specimens for genome sequencing, while minimizing the sequence diversity, an experimental lineage was set up in 2021 from a single parasite. The host populations of juvenile goldfish were procured from a fish market on multiple occasions. After morphologically identifying a gyrodactylid (*G. kobayashii*) on one goldfish specimen, the remaining goldfish specimens were treated with formaldehyde (concentration = 0.077 ppm) to remove ectoparasites, and reared in a fish tank in the laboratory. A single gyrodactylid parasite was removed from the tailfin of the infected goldfish specimen using tweezers and moved to a parasite‐free goldfish specimen (anaesthetized with MS‐222). After confirming attachment of the parasite, the infected specimen was placed in a separate tank (2 L volume), reared for a week alone, and then inspected for the presence of gyrodactylids. When their number on a single fish specimen exceeded five, a single parasite was detached, and identified morphologically and using an *18S* fragment (Supplementary file 1: Figure S6, Supporting Information). If the parasite was identified as *G. kobayashii*, the fish was moved to a fish tank (180 L volume) with hundreds of parasite‐free goldfish to further expand the population. This cycle was repeated multiple times. The fish in the 180‐L tank were regularly visually inspected, and when a fish specimen was identified as infected by a sufficiently high number of parasites (visual estimate ≥100 parasites), then tranquilized and placed it in hydrogen peroxide (30 microliters of 30% hydrogen peroxide per liter of water) to force parasites to fall off. After removing the fish, the remaining liquid was centrifuged to collect the parasites, followed by flash‐freezing them alive in liquid nitrogen, and finally stored them at −80 °C. To maximize the number of collected specimens and the amount of DNA, this cycle of periodical sampling of parasites was repeated over the period of six months, until ≈22000 *G. kobayashii* specimens were accumulated. They were finally divided into three clusters: ≈12000 for PacBio HiFi sequencing, ≈8000 for Hi‐C sequencing, and ≈2000 for transcriptome sequencing. All animal experiments were approved and conducted in compliance with the experimental practices and standards of the Ethics Committee of the College of Ecology, Lanzhou University (ethics approval form No. EAF2024012).

### DNA Extraction, Library Preparation, and Sequencing

Both genomic haplotypes of *G. kobayashii* were sequenced and assembled, using PacBio HiFi sequencing^[^ ^113^ ^]^ and Hi‐C sequencing.^[^ ^114^ ^]^ For PacBio HiFi sequencing, genomic DNA was extracted from pooled ≈12000 *G. kobayashii* specimens using the CTAB method and purified using the QIAGEN Genomic kit for regular sequencing according to the standard operating procedure provided by the manufacturer. The DNA degradation and contamination (protein+RNA) of the extracted DNA were checked on 1% agarose gels, and DNA purity was further assessed using the NanoDropTM One UV‐Vis spectrophotometer (Thermo Fisher Scientific, USA). OD260/280 values were between 1.8 and 2.0 and OD 260/230 values were between 2.0 and 2.2. DNA concentration was measured using the Qubit 4.0 Fluorometer (Invitrogen, USA). SMRTbell target size libraries were constructed for sequencing according to the PacBio's standard protocol (Pacific Biosciences, CA, USA) using 15 kb preparation solutions. The library preparation was conducted in six main steps: 1) DNA shearing by g‐TUBEs (Covaris, USA); 2) Single‐strand overhangs removal, followed by DNA damage repair, end repair, and A‐tailing; 3) Ligation of fragments with hairpin adapters from the SMRTbell Express Template Prep Kit 2.1 (Pacific Biosciences); 4) Nuclease treatment of SMRTbell library with SMRTbell Enzyme Cleanup Kit, target fragment screening by the BluePippin (Sage Science, USA), and purification of the SMRTbell library by AMPure PB beads; 5) Size selection, conducted using Agilent 2100 Bioanalyzer (Agilent Technologies, USA); 6) Sequencing, performed on a PacBio Sequel II instrument with Sequencing Primer V2 and Sequel II Binding Kit 2.1 in Haorui Genomics (Xi'an, China). Finally, the sequenced raw data were filtered to obtain a total of 15.63 GB of clean data, with the HiFi Read Quality (median) = Q33.

For Hi‐C sequencing, ≈8000 *G. kobayashii* specimens were subjected to formaldehyde crosslinking and Hi‐C library construction. DNA samples were digested using restriction endonucleases to assess cleavage efficiency. Following biotinylation, end‐repair, and biotin‐removal steps, Hi‐C libraries were prepared, and DNA quality was assessed. After the quality check, standard libraries were constructed. The procedure involved biotin removal from Hi‐C fragments, followed by sonication, end‐repair, A‐tailing, and adapter ligation. Subsequently, PCR conditions were optimized to amplify the library fragments, which were then extracted and subjected to quality control analysis for Hi‐C junctions. Sequencing was performed using the Illumina NovaSeq platform.

Transcriptome sequencing was conducted as described in the recent study,^[^ ^115^ ^]^ so details are provided in Supplementary file 1: Text S1. Briefly, the total RNA was extracted from ≈2000 *G. kobayashii* specimens, and sequenced using the Illumina HiSeq 4000 platform. The data were processed using FASTP,^[^ ^116^ ^]^ and assembled using Trinity.^[^ ^117^ ^]^ The open reading frame (ORF) prediction and translation were conducted in TransDecoder (https://github.com/TransDecoder). The raw genome and transcriptome data for *G. kobayashii* are available under the NCBI's BioProject number PRJNA1168307.

### Genome Assembly and Host DNA Contamination Removal

The HiFiasm assembler was used to assemble two genomic haplotypes (hap1 and hap2 henceforth).^[^ ^118^ ^]^ HapHiC^[^ ^119^ ^]^ was used for scaffolding, BWA‐MEM^[^ ^120^ ^]^ to index the contig‐level genome, and Juicer^[^ ^121^ ^]^ to create restriction enzyme cutting sites. In addition, to identify the ends of chromosomes, the distribution of telomere repeat sequences in the assembled genome was detected based on the vertebrate telomere sequence information using HapHiC's built‐in script. Given that gyrodactylids feed on the host's mucus and epithelial cells,^[^ ^20^ ^]^ the body surface and the gut of these parasites will invariably contain host cells and bacterial flora of the host's skin. To address this DNA pollution problem, a multi‐step contig filtering procedure of the processed Hi‐C data was implemented, aimed at confirming that all retained contigs exhibit an interaction with other contigs (i.e., that they belong to the genome of *G. kobayashii*): 1) BlobTools^[^ ^122^ ^]^ was used to filter contaminated contigs, via alignment of sequences against the UniProt UniRef90 database, and visualize sequencing depth from Hi‐C data. 2) All contigs were removed with low sequencing depth in Hi‐C data, while retaining the contigs annotated to Platyhelminthes in the UniProt UniRef90 database, presuming that these contigs are the genomic DNA of *G. kobayashii*. 3) The remaining contigs were assembled using HapHiC (Zeng et al.,2023) and visualized in Juicebox.^[^ ^123^ ^]^ Subsequently, Interactions were examined between individual contigs and primary contigs (contigs comprising the assembled haplotypes mentioned above). If no significant interactions were observed, the queried contig was removed. 4) It also aligned all contigs against the host (goldfish) genome.^[^ ^36^ ^]^ If a contig aligned well to the goldfish genome (minimap2 ‐cx asm20, contigs distributed diagonally) and did not show any interactions with the primary contigs in Juicebox, it was considered to be a host genome contamination and subsequently removed. 5) The remaining contigs were compared between the two haplotypes, and it was confirmed that they could be aligned to each other.

The run‐ASM‐pipeline.sh script of 3D‐DNA was utilized to scaffold a draft reference genome, and an assembly heatmap was generated using 3D‐DNA.^[^ ^124^ ^]^ Juicebox was used to manually correct assembly errors. The final assembly was obtained by using run‐ASM‐pipeline‐post‐review.sh script again to revise the results of the modified file output by Juicebox. The completeness of the assembly was assessed through BUSCO analysis.^[^ ^125^ ^]^

### Identification of Repeat Elements

Repeat elements were identified by running the pipeline Extensive de‐novo TE Annotator (EDTA).^[^ ^126^ ^]^ RepeatMasker and its RepeatProteinMask script^[^ ^127^ ^]^ were used to mask the whole genome sequences using the EDTA‐constructed TE library before gene prediction, as well as to identify known repeat element types by searching against the Repbase database. Tandem repeats were further ab initio predicted using the TRF tool.^[^ ^128^ ^]^

### Identification of SNPs

To assess the homozygosity of the culture system of *G. kobayashii*, a single‐nucleotide polymorphism (SNP) was called with the original HiFi sequencing reads and the two haplotype‐resolved reference genomes (hap1 and hap2). The raw reads were first aligned to the hap1/hap2 assemblies using Minimap2 v2.17‐r941,^[^ ^129^ ^]^ with parameters optimized for high‐accuracy long reads (‐ax map‐pb ‐asm5). The resulting alignment files were then converted from SAM format to BAM format and subsequently sorted using SAMtools v0.1.20.^[^ ^130^ ^]^ SNP calling was conducted using BCFtools v1.21.^[^ ^130^ ^]^ First, a pileup was generated from the sorted BAM file (mpileup command), and SNPs were then called using the call module with the multiallelic caller (‐mv), and filtered using the filter module to exclude low‐quality variants, specifically those with QUAL scores below 20 or depth (DP) greater than 100 (%QUAL<20 || DP>100). To quantify the level of homozygosity, the total number of SNPs identified in hap1/hap2 was calculated and expressed as a proportion of the total genome size. Genome lengths were computed using the stats command from SeqKit v2.0.0.^[^ ^131^ ^]^

### Gene Prediction and Annotation

The MAKER genome annotation pipeline^[^ ^132^ ^]^ was used to annotate the *G. kobayashii* genome assembly. MAKER coordinates several other applications, thus producing genome annotation from multiple lines of evidence. The custom repeat library created by EDTA (see previous section) was used by RepeatMasker within the MAKER pipeline to mask repetitive elements. MAKER then reconciled the assembled transcriptome sequences (inferred using Trinity, see above), homology‐based evidence, and the results of purely ab initio predicted gene models obtained using the following gene prediction tools: GeneMark,^[^ ^133^ ^]^ SNAP,^[^ ^134^ ^]^ and Augustus.^[^ ^135^ ^]^ For homology‐based prediction, the protein repertoires of *G. bullatarudis* and *G. salaris* were aligned against the *G. salaris* genome using MAKER plugins. The MAKER genome annotation pipeline was run three consecutive times, and the predicted gene models in each MAKER run were used for training gene prediction software programs SNAP and AUGUSTUS. Finally, eggNOG^[^ ^136^ ^]^ was used to perform functional annotations of predicted protein‐coding genes of *G. kobayashii* and its host against the Gene Ontology (GO), KEGG, and PFAM databases.

### Phylogenetic Analyses

Phylogenies were inferred independently for parasites and hosts. To avoid duplication of data, only hap1 of *G. kobayashii* was used for phylogenetic analyses. As the genome of the fourth available Monopisthocotylea species, *Benedenia humboldti*, was too low in quality, *Protopolystoma xenopodis* (Polyopisthocotylea) was used as the outgroup. The dataset comprised 1040 single‐copy core orthologues identified across the four genomes (*G. kobayashii* hap1, *G. bullatarudis*, *G. salaris*, *P. xenopodis*). Due to the wide phylogenetic range of hosts (*C. auratus*, *P. reticulata*, *S. salar*), *Scleropages formosus* (Osteoglossomorpha), belonging to a sister lineage to all other Osteichthyes,^[^ ^137^ ^]^ was used as the outgroup, and 379 single‐copy core orthologues were used to infer the phylogeny. All single‐copy gene families were obtained using OrthoFinder, and phylogenetic analyses conducted using PhyloSuite v.1.2.3^[^ ^138^, ^139^ ^]^ and its plug‐in programs: sequences were aligned in batch in MAFFT,^[^ ^140^ ^]^ alignments trimmed using trimAI,^[^ ^141^ ^]^ concatenated by PhyloSuite (when required), the optimal evolutionary model inferred using ModelFinder,^[^ ^142^ ^]^ and the Maximum Likelihood phylogenetic analysis conducted using IQ‐TREE.^[^ ^143^ ^]^ To assess topological stability, the species tree was also inferred from gene trees using ASTRAL.^[^ ^144^ ^]^

### Identification of Potential Drug Targets

In silico identification of potential drug targets was conducted in the three gyrodactylid genomes following the methodology outlined by Xie et al.,^[^ ^145^ ^]^ To identify genes expected to be vital for the survival of the organism (named “lethal” henceforth), due to the absence of suitable data for flatworms, it relied on orthologues of genes linked to lethal‐gene‐knockdown *C. elegans* phenotypes, mined from the WormBase database:^[^ ^146^ ^]^ WormBase WS226: WBPhenotype:0000050, WBPhenotype:0000054, WBPhenotype:0000060, WBPhenotype:0000062, and sub‐phenotypes. Three criteria was further applied to compare host and parasite orthologues: query alignment length coverage (qcovs), subject alignment length coverage (scovs, inferred by dividing the alignment length – with gaps removed – by the length of the subject sequence), and sequence similarity (pident). Three datasets were generated by overlapping different sets of criteria. 1) “Lethal”: lethal genes that met the screening criteria: (pident≥50%) AND (qcovs≥50% OR scovs≥50%); 2) “ChEMBL”: genes with orthologues in the ChEMBL database^[^ ^147^ ^]^ that met the screening criteria: (pident≥50%) AND (qcovs≥50% OR scovs≥50%); and 3) “Parasite‐only”: genes that did not exhibit high similarity to host orthologues, i.e., genes that met the following screening criteria: (pident≤50%) OR (qcovs≤50% AND scovs≤50%).

To rank the identified potential drug target genes, they were assigned scores using the following Equations (1) and (2):^[^ ^145^ ^]^

(1) $$S_{\mathrm{target}}=\left(S_{l}+S_{c}\right)\times 2+S_{t}+S_{e}$$

(2) $$S_{t}=log\left(T\right)/log\left(10\right)$$

Herein, T represents gene expression level in transcripts per million (TPM); and S_l_ and S_c_ were calculated using the formula: S_l_ = S/L, S_c_ = S/L, where S represents the alignment score of a pair of proteins, L represents the subject sequence length, lower case l represents “lethal”, and lower case c represents “ChEMBL”. S_e_ equals 1 if the target gene encodes a protease, protein kinase, protein phosphorylase, transporter, or ion channel (putative enzymatic chokepoints); otherwise, S_e_ is 0. For the TPM data, it relied on the *G. kobayashii* transcriptome sequenced in this study, and *G. bullatarudis* transcriptome sequenced previously^[^ ^30^ ^]^ (as the expression data were not available for *G. salaris*). The analyses were conducted separately for each species, using only hap 1 for *G. kobayashii*.

Using the filtered gene sets introduced above, three rounds of identification were conducted, with different levels of stringency. (1) “ChEMBL” and “lethal” sets of genes were merged to generate the broadest selection of identified drug targets. (2) To increase the stringency, the subset of genes were further identified that overlapped between the “ChEMBL” and “lethal” datasets. (3) The overlap of “lethal” and “parasite‐only” datasets. This step was followed by a manual assessment of the presence of gene orthologues in public databases to avoid annotation artifacts.

### Evaluation of In Vitro and In Vivo Anthelmintic Drug Activity Against *G. kobayashii* and Acute Toxicity in Goldfish

The anthelmintic activity of candidate drugs (≥ 95% purity, all made by Shanghai Aladdin Bio‐Technology) against *G. kobayashii* and their acute toxicity to goldfish were evaluated following the methodology outlined before.^[^ ^26^, ^59^ ^]^ Treatment concentration gradients were established based on the reported IC_50_ value of each candidate drug: Atogepant (5 – 100 µM), Conivaptan (0.5 – 10 µM), Imatinib (2.5 – 50 µM), or 0.02% DMSO (control group).^[^ ^62^, ^148^, ^149^ ^]^ For in vitro assays, infected goldfish (>200 *G. kobayashii* per caudal fin) were collected from the monoxenic culture system and anaesthetized with MS‐222 (0.02%), and caudal fins were sampled. Caudal fin fragments (containing 2–10 parasites each) were incubated in 24‐well plates (500 µL per well) for 6 h, ensuring 24 replicates per group. *G. kobayashii* viability was assessed hourly under a stereomicroscope, with death defined by the absence of movement after the mechanical stimulation with a fine needle.

For in vivo assays, goldfish (n = 10/group; each specimen 40–200 parasites per fin, as determined by stereomicroscopic enumeration) were exposed to imatinib (12.5 – 50 µM) in aerated water (25 ± 1 °C). At 24 and 48 h post‐treatment, fish were anaesthetized with MS‐222 (50 mg L^−1^), and parasites counted under a stereomicroscope. Anthelmintic efficacy and EC_50_ values were calculated using Probit analysis (SPSS 20.0).^[^ ^150^, ^151^ ^]^

For acute host toxicity testing, healthy goldfish of similar size (5.0 ± 0.5 cm, n = 10/tank) were exposed to Imatinib (25 – 200 µM), 0.02% DMSO (solvent control), or aerated tap water (negative control). Mortality was recorded at 12‐h intervals throughout the 48‐h exposure period, and 48‐h LC_50_ values were determined using Probit analysis (SPSS 20.0).^[^ ^150^ ^]^

### Homology Modeling and Molecular Docking of *G. kobayashii* Innexin 13

The protein homology modeling was performed using the inferred amino acid sequence of *G. kobayashii* INX‐13 and the SWISS‐MODEL server^[^ ^152^ ^]^ using the *T. asiatica* (Platyhelminthes: Cestoda) innexin (unspecified paralogue, accession number: A0A158R9B3) as the template. The inferred protein model was then used to conduct molecular docking following the methodology described previously.^[^ ^59^ ^]^ Briefly, ligands, comprising FDA‐approved drugs, were retrieved from DrugBank^[^ ^153^ ^]^ and PubChem^[^ ^154^ ^]^ databases, pre‐filtered based on molecular weight and atom types, and converted to PDBQT format using Open Babel v3.1.1.^[^ ^155^ ^]^ Each compound was tested against the protein's active site via docking simulations, conducted using PyRx v0.8^[^ ^156^ ^]^ with integrated AutoDock Vina engine,^[^ ^157^ ^]^ using a grid box with dimensions of 22.5 × 11.0 × 20.5 Å, centered at x: 19.25, y: 4.0, z: ‐2.25 Å. Docking parameters were: exhaustiveness = 9, and the energy range = 3. Binding energies were calculated for all protein‐ligand interactions, where low (range spans 0) binding energies reflect strong binding affinity. The protein‐ligand complexes were visualized using PyMOL Molecular Graphics System (version 2.5.2)^[^ ^158^ ^]^ and BIOVIA Discovery Studio Visualizer (Dassault Systèmes, the 3DEXPERIENCE Company).

### Genome‐Wide Host‐Parasite Protein Interaction Analysis

The prediction of interacting proteins between gyrodactylids and their hosts primarily relied on the orthology transfer methodology employed previously,^[^ ^10^, ^15^ ^]^ which relies on a homology‐based inference strategy and infers Protein‐Protein Interaction Networks using the Functional Enrichment Analysis in the STRING database.^[^ ^14^ ^]^ For this, first identified the contact proteomes of the three fish hosts and secretomes of the three gyrodactylid parasites.

The contact proteomes of three selected fish hosts (*C. auratus*, *S. salar*, *and P. reticulata*) were predicted in three steps. First, protein‐coding genes expressed in the fish tissues that come in contact with gyrodactylids, such as fins, skin, gills, mucus, mouth, etc., were identified from the ZFIN database for the model fish, zebrafish (*Danio rerio*).^[^ ^159^ ^]^ Proteins located in the cell membrane and extracellular space (presumed to come into contact with parasites) were further identified using the DeepLoc program.^[^ ^160^ ^]^ The results were processed using TargetP^[^ ^161^ ^]^ to remove mitochondrial genes. Finally, the filtered dataset was queried against the genomes of the three host fish species using the Reciprocal BLAST method. The purpose of the last step was to identify homologous genes of these selected zebrafish genes in each host.

In the next step, secretomes of the three gyrodactylids (*G. kobayashii, G. bullatarudis*, and *G. salaris*) were predicted, using SecretomeP^[^ ^162^ ^]^ and SignalP^[^ ^163^ ^]^ tools. The results were processed using TargetP and TMHMM^[^ ^164^ ^]^ tools to remove mitochondrial and transmembrane proteins, respectively. This process ultimately yielded filtered secretomes for the three gyrodactylids. In order to predict protein‐protein interactions using zebrafish as a model in the STRING database, the reciprocal BLAST method was used to identify homologous genes between these pooled secretomes and the zebrafish proteome. Homologous protein‐coding genes identified in all four species (three parasites and zebrafish) were selected for downstream analyses.

The STRING database was used to predict the intra‐species protein interactions of zebrafish genes identified in the previous two steps (contactome and secretome). This database integrates evidence from seven parameters: experimental data, text mining, database sources, gene fusion, gene neighborhood, gene co‐expression, and gene co‐occurrence. Each type of evidence is assigned a specific weight using an intrinsic scoring mechanism, and the recalculated combined score was then used to filter for only high‐confidence interactions (> 0.7).^[^ ^15^ ^]^ After filtering the known protein interaction data for zebrafish, Reciprocal BLAST was employed to identify homologs and transfer the intraspecies PPI of zebrafish to the parasite‐host system. These extrapolated inter‐species interaction proteins of the host‐gyrodactylid system were referred to as interologs. Specifically, when the host protein A and the parasite protein A' are orthologous genes, and the host protein B and the parasite protein B' are orthologous genes as well, if A and B are known interacting proteins in zebrafish, it was hypothesized that A″‐B and A‐B″ are interologs (interacting proteins) between the parasite and the host. The STRING database^[^ ^165^ ^]^ was utilized to construct the interaction network of these proteins, and the betweenness centrality (a metric used to measure the importance and influence of nodes in a network) was used as an indicator to assess the importance of nodes in the interaction network. It then used NetworkX^[^ ^166^ ^]^ to visualise the interaction network of proteins. This approach allowed us to in silico predict the set of proteins that might be involved in the interaction between gyrodactylids and hosts. KOBAS (KEGG Orthology Based Annotation System) was used to functionally annotate the PPI genes and infer functional pathways, followed by a statistical analysis of pathway enrichment using clusterProfiler^[^ ^167^ ^]^ with contactome and secretome as the background gene sets.

This was further relied on the congruence of gene‐level phylogenetic trees as an indicator of coevolution between hosts and parasites.^[^ ^10^, ^67^ ^]^ This test was based on the observation that genomic phylogenies are not congruent between hosts and parasites (see Figure 3), and the proposition that coevolution of interacting proteins in host‐parasite systems may produce apparent phylogenetic affinity.^[^ ^67^ ^]^ This was tested by inferring gene phylogenies using all identified PPI pairs of genes, and calculating pairwise distances between the three parasites and three hosts. If parasites are A (*Gyrodactylus kobayashii*), B (*Gyrodactylus salaris*) and C (*Gyrodactylus bullatarudis*), and hosts are A’ (*Carassius auratus*), B’ (*Salmo salar*) and C’ (*Poecilia reticulata*), the species tree patterns are: A–B < A–C/B–C for parasites, and B’–C’ < A’–B’/A’–C’ for hosts. There are three possible topologies for each combination of three species: the “parasite species topology” (AB_C), the “host species topology” (BC_A), and the “random topology” (AC_B). When the branch distance patterns were congruent between the parasite and the host orthologue, the candidate orthologues were flagged as exhibiting cophylogenetic relationships, but only if they both conformed to the “parasite” or “host” species topologies: (1) AB_C: A–B<A–C/B–C ∧ A’–B’<A’–C’/B’–C’; and (2) BC_A: B–C<A–B/A–C ∧ B’–C’<A’–B’/A’–C’, where ∧ indicates that both conditions must be true. Pairs of genes exhibiting the “random” topology (AC_B) were not counted in the cophylogenetic dataset, as it is assumed that these may be accidental.

### Statistical Analysis

This was relied on one‐sided Two Proportion Z‐test module in Python (statsmodels.stats.proportion.proportions_ztest) to statistically test (Z‐value; p‐value) whether the proportion of genes not conforming to the species topology was increased among the interacting proteins (PPIs) on the parasite (n = 290) and host (n = 329) side in comparison to the same proportion among single‐copy orthologues used to infer the species trees (n_parasite_ = 1040; n_host_ = 379).

### Ethics Approval Statement

All animal experiments were approved and conducted in compliance with the experimental practices and standards of the Ethics Committee of the College of Ecology, Lanzhou University (ethics approval form No. EAF2024012).

## Conflict of Interest

The authors declare no conflict of interest

## Author Contributions

D.Z. performed conceptualization, data curation, formal analysis, funding acquisition, investigation, methodology, project administration, resources, supervision, validation, wrote – review & edited. J.M.Z. and C.Y.X. performed data curation, formal analysis, investigation, methodology, resources, wrote – review & edited. Y.W.M. performed data curation, formal analysis, investigation, methodology, resources, and visualization. H.P.L. performed conceptualization, data curation, formal analysis, investigation, methodology, resources, visualization, wrote original draft. Y.Y.S., S.Z., X.F.Z., and J.S.C. performed data curation, formal analysis, investigation, methodology, resources, visualization, wrote review & edited. F.L. and B.Z. performed data curation, formal analysis, investigation, resources, wrote– review & edited. R.S., Y.H., F.Z., and X.L. performed investigation, funding acquisition, methodology, validation, wrote review & edited. W.X.L. and G.T.W. performed conceptualization, resources, supervision, validation, wrote review & edited. I.J. performed conceptualization, funding acquisition, investigation, methodology, supervision, validation, wrote original draft, wrote review, and edited.

## Supporting information



Supporting Information

Supporting Information

## Data Availability

The data that support the findings of this study are openly available in the NCBI’s BioProject database at https://www.ncbi.nlm.nih.gov/bioproject/?term=PRJNA1168307, reference number PRJNA1168307, and in the NGDC database under the BioProject PRJCA033671, sample number SAMC4463400, and the Accession numbers: GWHFQJJ00000000.1 and GWHFIHX00000000.1 (https://ngdc.cncb.ac.cn/gwh/search/advanced/result?search_category=&search_term=&source=0&query_box=SAMC4463400).

## References

[1] L. J. Buckingham , B. Ashby , J. Evol. Biol. 2022, 35, 205. 35030276 10.1111/jeb.13981 PMC9305583

[2] A. Tellier , S. Moreno‐Gámez , W. Stephan , Evolution 2014, 68, 2211. 24749791 10.1111/evo.12427

[3] B. J. Z. Quigley , D. García López , A. Buckling , A. J. McKane , S. P. Brown , R. Soc. B Biol. Sci 2012, 279, 3742. 10.1098/rspb.2012.0769 PMC3415897 22740644

[4] R. N. Beech , P. Skuce , D. J. Bartley , R. J. Martin , R. K. Prichard , J. S. Gilleard , Parasitology 2011, 138, 160. 20825689 10.1017/S0031182010001198 PMC3064440

[5] P. McVeigh , Parasitology 2020, 147, 835. 32252832 10.1017/S0031182020000591 PMC7284816

[6] C. M. Taylor , K. Fischer , S. Abubucker , Z. Wang , J. Martin , D. Jiang , M. Magliano , M.‐N. Rosso , B.‐W. Li , P. U. Fischer , M. Mitreva , PLoS One 2011, 6, 18381. 10.1371/journal.pone.0018381 PMC3083401 21556146

[7] R. Osborne , L. Rehneke , S. Lehmann , J. Roberts , M. Altmann , S. Altmann , Y. Zhang , E. Köpff , A. Dominguez‐Ferreras , E. Okechukwu , C. Sergaki , C. Rich‐Griffin , V. Ntoukakis , R. Eichmann , W. Shan , P. Falter‐Braun , P. Schäfer , Nat. Commun. 2023, 14, 4065. 37429856 10.1038/s41467-023-39885-5 PMC10333260

[8] K. James , P. D. Olson , BMC Genomics 2020, 21, 346. 32380953 10.1186/s12864-020-6710-1 PMC7204028

[9] M. Hue , M. Riffle , J.‐P. Vert , W. S. Noble , BMC Bioinformatics 2010, 11, 144. 20298601 10.1186/1471-2105-11-144 PMC2845582

[10] Y. Hu , L. Yu , H. Fan , G. Huang , Q. Wu , Y. Nie , S. Liu , L. Yan , F. Wei , Mol. Biol. Evol. 2021, 38, 531. 32960966 10.1093/molbev/msaa243 PMC7826172

[11] International Helminth Genomes Consortium , Nat. Genet. 2019, 51, 163. 30397333 10.1038/s41588-018-0262-1 PMC6349046

[12] C. von Mering , E. M. Zdobnov , S. Tsoka , F. D. Ciccarelli , J. B. Pereira‐Leal , C. A. Ouzounis , P. Bork , Proc. Natl. Acad. Sci. USA 2003, 100, 15428. 14673105 10.1073/pnas.2136809100 PMC307584

[13] L. R. Matthews , P. Vaglio , J. Reboul , H. Ge , B. P. Davis , J. Garrels , S. Vincent , M. Vidal , Genome Res. 2001, 11, 2120. 11731503 10.1101/gr.205301 PMC311221

[14] D. Szklarczyk , A. Franceschini , S. Wyder , K. Forslund , D. Heller , J. Huerta‐Cepas , M. Simonovic , A. Roth , A. Santos , K. P. Tsafou , M. Kuhn , P. Bork , L. J. Jensen , C. V. Mering , Nucleic Acids Res. 2015, 43, D447. 25352553 10.1093/nar/gku1003 PMC4383874

[15] Y. Cuesta‐Astroz , A. Santos , G. Oliveira , L. J. Jensen , Immunol. 2019, 10, 212. 10.3389/fimmu.2019.00212 PMC6381214 30815000

[16] C. J. Carlson , T. A. Dallas , L. W. Alexander , A. L. Phelan , A. J. Phillips , Proc. R. Soc. B Biol. Sci. 2020, 287, 20201841. 10.1098/rspb.2020.1841 PMC7739500 33203333

[17] J. Brabec , E. D. Salomaki , M. Kolísko , T. Scholz , R. Kuchta , Curr. Biol. 2023, 33, 4269. 37729914 10.1016/j.cub.2023.08.064

[18] J.‐L. Justine , Int. J. Parasitol. 1998, 28, 1653. 9801923 10.1016/s0020-7519(98)00060-5

[19] P. D. Harris , A. P. Shinn , J. Cable , T. A. Bakke , Syst. Parasitol. 2004, 59, 1. 15318017 10.1023/B:SYPA.0000038447.52015.e4

[20] T. A. Bakke , J. Cable , P. D. Harris , in Advanced Parasitology, (Eds.: J.R. Baker , R. Muller , D. Rollinson ), Academic Press, Amsterdam, Netherlands, 2007, pp. 161.

[21] M. P. M. Vanhove , T. Huyse , In *Parasite*D*iversified Evolution Ecology Meets Phylogenetics* , (Eds.: B.R. Krasnov , D.T.J. Littlewood , S. Morand ), Cambridge University Press, Cambridge 2015, pp. 401.

[22] M. Konczal , A. R. Ellison , K. P. Phillips , J. Radwan , R. S. Mohammed , J. Cable , M. Chadzinska , Parasite Immunol. 2020, 42, 12782. 10.1111/pim.12782 32738163

[23] K. P. Phillips , J. Cable , R. S. Mohammed , M. Herdegen‐Radwan , J. Raubic , K. J. Przesmycka , C. van Oosterhout , J. Radwan , Natl. Acad. Sci. 2018, 115, 1552. 10.1073/pnas.1708597115 PMC5816137 29339521

[24] K. P. Phillips , J. Cable , R. S. Mohammed , S. Chmielewski , K. J. Przesmycka , C. van Oosterhout , J. Radwan , Mol. Ecol. 2021, 30, 5588. 34415650 10.1111/mec.16135 PMC9292977

[25] T. A. Bakke , P. D. Harris , J. Cable , Int. J. Parasitol. 2002, 32, 281. 11835970 10.1016/s0020-7519(01)00331-9

[26] S. Zhou , L. Xia , J. Dong , Y. Liu , Q. Yang , N. Xu , Y. Yang , X. Ai , Vet. Parasitol. 2023, 324, 110058. 39492189 10.1016/j.vetpar.2023.110058

[27] B. Schelkle , A. P. Shinn , E. Peeler , J. Cable , Dis. Aquat. Organ. 2009, 86, 65. 19899351 10.3354/dao02087

[28] X. Zhang , F. Wu , N. Yang , X. Zhan , J. Liao , S. Mai , Z. Huang , Interdiscip. Sci. Comput. Life Sci. 2022, 14, 285. 10.1007/s12539-021-00491-y PMC8616973 34826045

[29] C. Hahn , B. Fromm , L. Bachmann , Genome Biol. Evol. 2014, 6, 1105. 24732282 10.1093/gbe/evu078 PMC4040987

[30] M. Konczal , K. J. Przesmycka , R. S. Mohammed , K. P. Phillips , F. Camara , S. Chmielewski , C. Hahn , R. Guigo , J. Cable , J. Radwan , Mol. Ecol. 2020, 29, 1494. 32222008 10.1111/mec.15421

[31] S. Zhou , X. Jin , Q. Yang , J. Dong , Y. Liu , N. Xu , Y. Yang , X. Ai , Rep. 2022, 25, 101221.

[32] P. Monticini , The ornamental fish trade: production and commerce of ornamental fish: technical‐managerial and legislative aspects, Food and Agriculture Organization of the United Nations, Rome, Italy, 2010.

[33] X. Tu , F. Ling , A. Huang , G. Wang , Parasitol. Res. 2015, 114, 737. 25471903 10.1007/s00436-014-4241-x

[34] S. Zhou , W. X. Li , H. Zou , J. Zhang , S. G. Wu , M. Li , G. T. Wang , Fish Shellfish Immunol. 2018, 77, 40. 29567133 10.1016/j.fsi.2018.03.033

[35] J. Cable , P. D. Harris , Int. J. Parasitol. 2002, 32, 255. 11835969 10.1016/s0020-7519(01)00330-7

[36] Z. Chen , Y. Omori , S. Koren , T. Shirokiya , T. Kuroda , A. Miyamoto , H. Wada , A. Fujiyama , A. Toyoda , S. Zhang , T. G. Wolfsberg , K. Kawakami , A. M. Phillippy , N. C. S. Program , J. C. Mullikin , S. M. Burgess , Sci. Adv. 2019, 5, aav0547. 10.1126/sciadv.aav0547 PMC6594761 31249862

[37] A. Künstner , M. Hoffmann , B. A. Fraser , V. A. Kottler , E. Sharma , D. Weigel , C. Dreyer , PLoS One 2016, 11, 0169087. 10.1371/journal.pone.0169087 PMC5199103 28033408

[38] S. Lien , B. F. Koop , S. R. Sandve , J. R. Miller , M. P. Kent , T. Nome , T. R. Hvidsten , J. S. Leong , D. R. Minkley , A. Zimin , F. Grammes , H. Grove , A. Gjuvsland , B. Walenz , R. A. Hermansen , K. von Schalburg , E. B. Rondeau , A. Di Genova , J. K. A. Samy , J. Olav Vik , M. D. Vigeland , L. Caler , U. Grimholt , S. Jentoft , D. Inge Våge , P. de Jong , T. Moen , M. Baranski , Y. Palti , D. R. Smith , et al., Nature 2016, 533, 200. 27088604 10.1038/nature17164 PMC8127823

[39] V. Praitis , J. Simske , S. Kniss , R. Mandt , L. Imlay , C. Feddersen , M. B. Miller , J. Mushi , W. Liszewski , R. Weinstein , A. Chakravorty , D.‐G. Ha , A. S. Farrell , A. Sullivan‐Wilson , T. Stock , PLoS Genet. 2013, 9, 1003506. 10.1371/journal.pgen.1003506 PMC3656159 23696750

[40] E. Schifano , G. Ficociello , S. Vespa , S. Ghosh , J. F. Cipollo , C. Talora , L. V. Lotti , P. Mancini , D. Uccelletti , Virulence 2019, 10, 1013. 31771413 10.1080/21505594.2019.1697118 PMC6930020

[41] M. Verleih , A. Rebl , B. Köllner , T. Korytář , J. M. Köbis , C. Kühn , K. Wimmers , T. Goldammer , Gene 2013, 512, 251. 23137639 10.1016/j.gene.2012.10.037

[42] S. Rodrigue , R. R. Malmstrom , A. M. Berlin , B. W. Birren , M. R. Henn , S. W. Chisholm , PLoS One 2009, 4, 6864. 10.1371/journal.pone.0006864 PMC2731171 19724646

[43] I. J. Tsai , M. Zarowiecki , N. Holroyd , A. Garciarrubio , A. Sanchez‐Flores , K. L. Brooks , A. Tracey , R. J. Bobes , G. Fragoso , E. Sciutto , M. Aslett , H. Beasley , H. M. Bennett , J. Cai , F. Camicia , R. Clark , M. Cucher , N. De Silva , T. A. Day , P. Deplazes , K. Estrada , C. Fernández , P. W. H. Holland , J. Hou , S. Hu , T. Huckvale , S. S. Hung , L. Kamenetzky , J. A. Keane , F. Kiss , et al., Nature 2013, 496, 57. 23485966 10.1038/nature12031 PMC3964345

[44] P. Phelan , J. P. Bacon , J. A. Davies , L. A. Stebbings , M. G. Todman , Trends Genet. 1998, 14, 348. 9769729 10.1016/s0168-9525(98)01547-9 PMC4442478

[45] J. Güiza , I. Barría , J. C. Sáez , J. L. Vega , Front. Physiol. 2018, 9, 1414. 30364195 10.3389/fphys.2018.01414 PMC6193117

[46] R. C. Johnsen , S. J. M. Jones , A. M. Rose , Mol. Gen. Genet. MGG 2000, 263, 239. 10778742 10.1007/s004380051165

[47] T. Starich , M. Sheehan , J. Aho , J. Shaw , Cell Commun. Adhes. 2001, 8, 311. 12064609 10.3109/15419060109080744

[48] J. S.‐C. Chu , S.‐Y. Chua , K. Wong , A. M. Davison , R. Johnsen , D. L. Baillie , A. M. Rose , BMC Genomics 2014, 15, 361. 24884423 10.1186/1471-2164-15-361 PMC4039747

[49] J. Chu , R. Johnsen , S. Chua , D. Tu , M. Dennison , M. Marra , S. Jones , D. Baillie , A. Rose , Genetics 2012, 190, 1225. 22267497 10.1534/genetics.111.137208 PMC3316639

[50] F. Simmer , C. Moorman , A. M. van der Linden , E. Kuijk , P. V. E. van den Berghe , R. S. Kamath , A. G. Fraser , J. Ahringer , R. H. A. Plasterk , PLoS Biol. 2003, 1, 12. 10.1371/journal.pbio.0000012 PMC212692 14551910

[51] J. Ceron , J.‐F. Rual , A. Chandra , D. Dupuy , M. Vidal , S. van den Heuvel , BMC Dev. Biol. 2007, 7, 30. 17417969 10.1186/1471-213X-7-30 PMC1863419

[52] L. Molin , H. Schnabel , T. Kaletta , R. Feichtinger , I. A. Hope , R. Schnabel , Genetics 1999, 151, 131. 9872954 10.1093/genetics/151.1.131 PMC1460461

[53] S. Kumar , K. Chaudhary , J. M. Foster , J. F. Novelli , Y. Zhang , S. Wang , D. Spiro , E. Ghedin , C. K. S. Carlow , PLoS One 1189, 2007, 2. 10.1371/journal.pone.0001189 PMC2063515 18000556

[54] R. Bergquist , J. Utzinger , J. Keiser , Infect. Dis. Poverty 2017, 6, 74. 28351414 10.1186/s40249-017-0286-2 PMC5371198

[55] J. Kang , N. Brajanovski , K. T. Chan , J. Xuan , R. B. Pearson , E. Sanij , Signal Transduct. Target. Ther. 2021, 6, 323. 34462428 10.1038/s41392-021-00728-8 PMC8405630

[56] X. Zhou , W.‐J. Liao , J.‐M. Liao , P. Liao , H. Lu , J. Mol. Cell Biol. 2015, 7, 92. 25735597 10.1093/jmcb/mjv014 PMC4481666

[57] A. Dillin , A.‐L. Hsu , N. Arantes‐Oliveira , J. Lehrer‐Graiwer , H. Hsin , A. G. Fraser , R. S. Kamath , J. Ahringer , C. Kenyon , Science 2002, 298, 2398. 12471266 10.1126/science.1077780

[58] M. L. LaBella , E. J. Hujber , K. A. Moore , R. L. Rawson , S. A. Merrill , P. D. Allaire , M. Ailion , J. Hollien , M. J. Bastiani , E. M. Jorgensen , Dev. Cell 2020, 52, 88. 31910362 10.1016/j.devcel.2019.12.005 PMC7263923

[59] J. Dong , L. Xia , Y. Liu , Q. Yang , N. Xu , X. Ai , S. Zhou , J. Fish Dis. 2025, 48, 14102. 10.1111/jfd.14102 39992024

[60] G. Sliwoski , S. Kothiwale , J. Meiler , E. W. Lowe , Pharmacol. Rev. 2014, 66, 334. 24381236 10.1124/pr.112.007336 PMC3880464

[61] B. Peng , P. Lloyd , H. Schran , Clin. Pharmacokinet. 2005, 44, 879. 16122278 10.2165/00003088-200544090-00001

[62] S. Hemer , K. Brehm , J. Antimicrob. Agents 2012, 40, 458. 10.1016/j.ijantimicag.2012.07.007 22947125

[63] Y. Liu , X. Tan , Y. Zhang , F. Ling , T. Liu , G. Wang , Aquaculture 2022, 560, 738552.

[64] X. Tu , C. Duan , S. Wu , S. Fu , J. Ye , Aquaculture 2021, 535, 736372.

[65] R. M. Maizels , H. H. Smits , H. J. McSorley , Immunity 2018, 49, 801. 30462997 10.1016/j.immuni.2018.10.016 PMC6269126

[66] M. Salzet , A. Capron , G. B. Stefano , Today 2000, 16, 536. 10.1016/s0169-4758(00)01787-7 11121852

[67] S. C. Lovell , D. L. Robertson , Biol. Evol 2010, 27, 2567. 10.1093/molbev/msq144 20551042

[68] T. D. L. Campos , N. D. Young , P. K. Korhonen , R. S. Hall , S. Mangiola , A. Lonie , R. B. Gasser , Parasit. Vectors 2014, 7, 242. 24884876 10.1186/1756-3305-7-242 PMC4100253

[69] S. Braschi , R. S. Curwen , P. D. Ashton , S. Verjovski‐Almeida , A. Wilson , Proteomics 2006, 6, 1471. 16447162 10.1002/pmic.200500368

[70] D. C. New , J. T. Y. Wong , Biol. Signals Recept. 1998, 7, 98. 9629461 10.1159/000014535

[71] M. C. Lagerström , H. B. Schiöth , Nat. Rev. Drug Discovery 2008, 7, 339. 18382464 10.1038/nrd2518

[72] E. Stefan , M. K. Malleshaiah , B. Breton , P. H. Ear , V. Bachmann , M. Beyermann , M. Bouvier , S. W. Michnick , Nat. Commun. 2011, 2, 598. 22186894 10.1038/ncomms1605 PMC3247815

[73] W. Harnett , M. M. Harnett , Parasitol. Today 1998, 14, 27. 17040686 10.1016/s0169-4758(97)01167-8

[74] T. Harrison , B. U. Samuel , T. Akompong , H. Hamm , N. Mohandas , J. W. Lomasney , K. Haldar , Science 2003, 301, 1734. 14500986 10.1126/science.1089324

[75] E. Lasonder , K. More , S. Singh , M. Haidar , D. Bertinetti , E. J. Kennedy , F. W. Herberg , A. A. Holder , G. Langsley , C. E. Chitnis , Front. Microbiol. 2021, 12, 684005. 34108954 10.3389/fmicb.2021.684005 PMC8183823

[76] P. Phan , D. Liang , M. Zhao , R. C. Wyeth , C. Fogarty , M. G. Duke , D. P. McManus , T. Wang , S. F. Cummins , Sci. Rep. 2022, 12, 8243. 35581232 10.1038/s41598-022-11996-x PMC9114394

[77] J. M. Fitzpatrick , E. Peak , S. Perally , I. W. Chalmers , J. Barrett , T. P. Yoshino , A. C. Ivens , K. F. Hoffmann , PLoS Negl. Trop. Dis. 2009, 3, 543. 10.1371/journal.pntd.0000543 PMC2764848 19885392

[78] D. E. Bosch , D. P. Siderovski , Exp. Mol. Med. 2013, 45, 15. 10.1038/emm.2013.30 PMC3641396 23519208

[79] R. Santos , O. Ursu , A. Gaulton , A. P. Bento , R. S. Donadi , C. G. Bologa , A. Karlsson , B. Al‐Lazikani , A. Hersey , T. I. Oprea , J. P. Overington , Nat. Rev. Drug Discovery 2017, 16, 19. 27910877 10.1038/nrd.2016.230 PMC6314433

[80] A. S. Taft , F. A. Norante , T. P. Yoshino , Parasitol. 2010, 125, 84. 10.1016/j.exppara.2009.12.021 PMC2859107 20060828

[81] C. A. Stratakis , J. Pathol. 2018, 244, 257. 29205368 10.1002/path.5014

[82] H. Hu , Q. Li , L. Jiang , Y. Zou , J. Duan , Z. Sun , Toxicol. Res. 2016, 5, 609. 10.1039/c5tx00383k PMC6062350 30090375

[83] R. J. Evans , V. Sundaramurthy , E.‐M. Frickel , Cell Dev. Biol. 2018, 6, 118. 10.3389/fcell.2018.00118 PMC6146372 30271774

[84] S. Besteiro , Curr. Opin. Microbiol. 2017, 40, 14. 29096193 10.1016/j.mib.2017.10.008

[85] E. R. James , D. R. Green , Trends Parasitol 2004, 20, 280. 15147679 10.1016/j.pt.2004.04.004

[86] J. S. C. Arthur , S. C. Ley , Nat. Rev. Immunol. 2013, 13, 679. 23954936 10.1038/nri3495

[87] X. Tu , X. Qi , A. Huang , F. Ling , G. Wang , Fish Shellfish Immunol 2019, 86, 116. 30448448 10.1016/j.fsi.2018.11.035

[88] S. Zhou , Y. Liu , J. Dong , Q. Yang , N. Xu , Y. Yang , Z. Gu , X. Ai , Parasitol. Res. 2021, 120, 161. 33094386 10.1007/s00436-020-06827-9

[89] L. Wei , R. Zhang , J. Zhang , J. Li , D. Kong , Q. Wang , J. Fang , L. Wang , Mucosal Immunol. 2021, 14, 1282. 34349238 10.1038/s41385-021-00426-2 PMC8528707

[90] E. Saloustros , P. Salpea , C.‐F. Qi , L. A. Gugliotti , K. Tsang , S. Liu , M. F. Starost , H. C. Morse , C. A. Stratakis , J. Exp. Clin. Cancer Res. 2015, 34, 143. 26608815 10.1186/s13046-015-0257-z PMC4660639

[91] D. Kong , Y. Shen , G. Liu , S. Zuo , Y. Ji , A. Lu , M. Nakamura , M. Lazarus , C. A. Stratakis , R. M. Breyer , Y. Yu , J. Exp. Med. 2016, 213, 2209. 27621415 10.1084/jem.20160459 PMC5030806

[92] M. Afrin , H. Kishmiri , R. Sandhu , M. A. G. Rabbani , B. Li , mSphere 2020, 5, 10. 10.1128/mSphere.00027-20 PMC7045384 32102938

[93] N. Minato , K. Kohei , M. Hattori , Adv. Immunol. 2007, 93, 229. 17383543 10.1016/S0065-2776(06)93006-5

[94] V. Pizon , M. Desjardins , C. Bucci , R. G. Parton , M. Zerial , J. Cell Sci. 1994, 107, 1661. 7962206 10.1242/jcs.107.6.1661

[95] M. Hoshijima , A. Kikuchi , M. Kawata , T. Ohmori , E. Hashimoto , H. Yamamura , Y. Takai , Biochem. Biophys. Res. Commun. 1988, 157, 851. 2849942 10.1016/s0006-291x(88)80953-7

[96] J. Kaur , S. Dutta , K.‐P. Chang , N. Singh , J. Antimicrob. Chemother. 2013, 68, 1071. 23292345 10.1093/jac/dks507 PMC3625431

[97] D. Chakrabarti , T. Azam , C. DelVecchio , L. Qiu , Y. Park , C. M. Allen , Mol. Biochem. Parasitol. 1998, 94, 175. 9747968 10.1016/s0166-6851(98)00065-6

[98] G. Ferri , D. Musikant , M. M. Edreira , PLoS Negl. Trop. Dis. 2023, 17, 0011191. 10.1371/journal.pntd.0011191 PMC10032529 36897926

[99] E.‐J. Lee , C. Tournier , Autophagy 2011, 7, 689. 21460635 10.4161/auto.7.7.15450 PMC3149696

[100] G. Ghartey‐Kwansah , F. Adu‐Nti , B. Aboagye , A. Ankobil , E. E. Essuman , Y. K. Opoku , S. Abokyi , E. K. Abu , J. N. Boampong , Cell Biosci. 2020, 10, 101. 32944216 10.1186/s13578-020-00464-6 PMC7487832

[101] B. Frank , A. Marcu , A. L. de Oliveira Almeida Petersen , H. Weber , C. Stigloher , J. C. Mottram , C. J. Scholz , U. Schurigt , Parasit. Vectors 2015, 8, 404. 26226952 10.1186/s13071-015-0974-3 PMC4521392

[102] V. Deretic , T. Saitoh , S. Akira , Nat. Rev. Immunol. 2013, 13, 722. 24064518 10.1038/nri3532 PMC5340150

[103] Y. V. Budovskaya , S. Js , R. F. , K. Dj , H. Pk , J. Biol. Chem. 2004, 279, p20663. 10.1074/jbc.M400272200 PMC1705971 15016820

[104] Y. Nakase , T. Matsumoto , J. Cell Sci. 2018, 131, jcs221457. 30301783 10.1242/jcs.221457

[105] M. Zhang , J. Zhang , S.‐C. Lin , A. Meng , Development 2012, 139, 2009. 22535411 10.1242/dev.074435

[106] H. He , M.‐P. Brenier‐Pinchart , L. Braun , A. Kraut , B. Touquet , Y. Couté , I. Tardieux , M.‐A. Hakimi , A. Bougdour , eLife 2018, 7, 39887. 10.7554/eLife.39887 PMC6214654 30320549

[107] M. Jin , B. Yu , W. Zhang , W. Zhang , Z. Xiao , Z. Mao , Y. Lai , D. Lin , Q. Ma , E. Pan , Y. Zhang , Y. Yu , Integr. Biol. 2016, 8, 968. 10.1039/c6ib00097e 27515449

[108] I. Jakovlić , Y. B. Zhang , Q. J. Wu , J. F. Gui , Croat. J. Fish. 2006, 64, 65.

[109] Z. Kanwal , G. F. Wiegertjes , W. J. Veneman , A. H. Meijer , H. P. Spaink , Dev. Comp. Immunol. 2014, 46, 35. 24560981 10.1016/j.dci.2014.02.003

[110] R. F. Lai , I. Jakovlić , H. Liu , J. Wei , F. B. Zhan , P. H. Yang , W. M. Wang , J. Fish Biol. 2017, 90, 803. 27943292 10.1111/jfb.13199

[111] I. Masuho , H. Wakasugi‐Masuho , E. N. Posokhova , J. R. Patton , K. A. Martemyanov , J. Biol. Chem. 2011, 286, 21806. 21511947 10.1074/jbc.M111.241513 PMC3122235

[112] M. J. Gilhooley , D. G. Hickey , M. Lindner , T. Palumaa , S. Hughes , S. N. Peirson , R. E. MacLaren , M. W. Hankins , Exp. Eye Res. 2021, 207, 108553. 33811915 10.1016/j.exer.2021.108553 PMC8214074

[113] A. M. Wenger , P. Peluso , W. J. Rowell , P.‐C. Chang , R. J. Hall , G. T. Concepcion , J. Ebler , A. Fungtammasan , A. Kolesnikov , N. D. Olson , A. Töpfer , M. Alonge , M. Mahmoud , Y. Qian , C.‐S. Chin , A. M. Phillippy , M. C. Schatz , G. Myers , M. A. DePristo , J. Ruan , T. Marschall , F. J. Sedlazeck , J. M. Zook , H. Li , S. Koren , A. Carroll , D. R. Rank , M. W. Hunkapiller , Nat. Biotechnol. 2019, 37, 1155. 31406327 10.1038/s41587-019-0217-9 PMC6776680

[114] J.‐M. Belton , R. P. McCord , J. H. Gibcus , N. Naumova , Y. Zhan , J. Dekker , Methods 2012, 58, 268. 22652625 10.1016/j.ymeth.2012.05.001 PMC3874846

[115] D. Zhang , I. Jakovlić , H. Zou , F. Liu , C.‐Y. Xiang , Q. Gusang , S. Tso , S. Xue , W.‐J. Zhu , Z. Li , J. Wu , G.‐T. Wang , Int. J. Parasitol. 2024, 54, 213. 38185351 10.1016/j.ijpara.2024.01.001

[116] S. Chen , Y. Zhou , Y. Chen , J. Gu , Bioinformatics 2018, 34, i884. 30423086 10.1093/bioinformatics/bty560 PMC6129281

[117] M. G. Grabherr , B. J. Haas , M. Yassour , J. Z. Levin , D. A. Thompson , I. Amit , X. Adiconis , L. Fan , R. Raychowdhury , Q. Zeng , Z. Chen , E. Mauceli , N. Hacohen , A. Gnirke , N. Rhind , F. di Palma , B. W. Birren , C. Nusbaum , K. Lindblad‐Toh , N. Friedman , A. Regev , Nat. Biotechnol. 2011, 29, 644. 21572440 10.1038/nbt.1883 PMC3571712

[118] H. Cheng , G. T. Concepcion , X. Feng , H. Zhang , H. Li , Nat. Methods 2021, 18, 170. 33526886 10.1038/s41592-020-01056-5 PMC7961889

[119] X. Zeng , Z. Yi , X. Zhang , Y. Du , Y. Li , Z. Zhou , S. Chen , H. Zhao , S. Yang , Y. Wang , G. Chen , Nat. Plants 2024, 10, 1184. 39103456 10.1038/s41477-024-01755-3

[120] H. Li , arXiv 2013, 13033997.

[121] N. C. Durand , M. S. Shamim , I. Machol , S. S. P. Rao , M. H. Huntley , E. S. Lander , E. L. Aiden , Cell Syst. 2016, 3, 95. 27467249 10.1016/j.cels.2016.07.002 PMC5846465

[122] D. R. Laetsch , M. L. Blaxter , F1000Research 2017, 122321.

[123] N. C. Durand , J. T. Robinson , M. S. Shamim , I. Machol , J. P. Mesirov , E. S. Lander , E. L. Aiden , Cell Syst. 2016, 3, 99. 27467250 10.1016/j.cels.2015.07.012 PMC5596920

[124] O. Dudchenko , S. S. Batra , A. D. Omer , S. K. Nyquist , M. Hoeger , N. C. Durand , M. S. Shamim , I. Machol , E. S. Lander , A. P. Aiden , E. L. Aiden , Science 2017, 356, 92. 28336562 10.1126/science.aal3327 PMC5635820

[125] M. Manni , M. R. Berkeley , M. Seppey , E. M. Zdobnov , Curr. Protoc. 2021, 1, 323. 10.1002/cpz1.323 34936221

[126] S. Ou , W. Su , Y. Liao , K. Chougule , J. R. A. Agda , A. J. Hellinga , C. S. B. Lugo , T. A. Elliott , D. Ware , T. Peterson , N. Jiang , C. N. Hirsch , M. B. Hufford , Genome Biol. 2019, 20, 275. 31843001 10.1186/s13059-019-1905-y PMC6913007

[127] M. Tarailo‐Graovac , N. Chen , Curr. Protoc. Bioinforma. 2009, 25, 4101. 10.1002/0471250953.bi0410s25 19274634

[128] G. Benson , Nucleic Acids Res. 1999, 27, 573. 9862982 10.1093/nar/27.2.573 PMC148217

[129] H. Li , Bioinformatics 2018, 34, 3094. 29750242 10.1093/bioinformatics/bty191 PMC6137996

[130] P. Danecek , J. K. Bonfield , J. Liddle , J. Marshall , V. Ohan , M. O. Pollard , A. Whitwham , T. Keane , S. A. McCarthy , R. M. Davies , H. Li , GigaScience 2021, 10, giab008. 33590861 10.1093/gigascience/giab008 PMC7931819

[131] W. Shen , B. Sipos , L. Zhao , iMeta 2024, 3, 191. 10.1002/imt2.191 PMC11183193 38898985

[132] B. L. Cantarel , I. Korf , S. M. C. Robb , G. Parra , E. Ross , B. Moore , C. Holt , A. S. Alvarado , M. Yandell , Genome Res. 2008, 18, 188. 18025269 10.1101/gr.6743907 PMC2134774

[133] A. V. Lukashin , M. Borodovsky , Nucleic Acids Res. 1998, 26, 1107. 9461475 10.1093/nar/26.4.1107 PMC147337

[134] A. D. Johnson , R. E. Handsaker , S. L. Pulit , M. M. Nizzari , C. J. O'Donnell , P. I. W. de Bakker , Bioinformatics 2008, 24, 2938. 18974171 10.1093/bioinformatics/btn564 PMC2720775

[135] M. Stanke , O. Keller , I. Gunduz , A. Hayes , S. Waack , B. Morgenstern , Nucleic Acids Res. 2006, 34, W435. 16845043 10.1093/nar/gkl200 PMC1538822

[136] C. P. Cantalapiedra , A. Hernández‐Plaza , I. Letunic , P. Bork , J. Huerta‐Cepas , Mol. Biol. Evol. 2021, 38, 5825. 34597405 10.1093/molbev/msab293 PMC8662613

[137] R. Betancur‐R , E. O. Wiley , G. Arratia , A. Acero , N. Bailly , M. Miya , G. Lecointre , G. Ortí , BMC Evol. Biol. 2017, 17, 162. 28683774 10.1186/s12862-017-0958-3 PMC5501477

[138] C. Xiang , F. Gao , I. Jakovlić , H. Lei , Y. Hu , H. Zhang , H. Zou , G. T. Wang , D. Zhang , iMeta 2023, 2, 87. 10.1002/imt2.87 PMC10989932 38868339

[139] D. Zhang , F. Gao , I. Jakovlić , H. Zou , J. Zhang , W. X. Li , G. T. Wang , Mol. Ecol. Resour. 2020, 20, 348. 31599058 10.1111/1755-0998.13096

[140] K. Katoh , D. M. Standley , Mol. Biol. Evol. 2013, 30, 772. 23329690 10.1093/molbev/mst010 PMC3603318

[141] S. Capella‐Gutiérrez , J. M. Silla‐Martínez , T. Gabaldón , Bioinformatics 2009, 25, 1972. 19505945 10.1093/bioinformatics/btp348 PMC2712344

[142] S. Kalyaanamoorthy , B. Q. Minh , T. K. F. Wong , A. Von Haeseler , L. S. Jermiin , Nat. Methods 2017, 14, 587. 28481363 10.1038/nmeth.4285 PMC5453245

[143] B. Q. Minh , H. A. Schmidt , O. Chernomor , D. Schrempf , M. D. Woodhams , A. von Haeseler , R. Lanfear , Mol. Biol. Evol. 2020, 37, 1530. 32011700 10.1093/molbev/msaa015 PMC7182206

[144] C. Zhang , M. Rabiee , E. Sayyari , S. Mirarab , BMC Bioinformatics 2018, 19, 153. 29745866 10.1186/s12859-018-2129-y PMC5998893

[145] Y. Xie , S. Wang , S. Wu , S. Gao , Q. Meng , C. Wang , J. Lan , L. Luo , X. Zhou , J. Xu , X. Gu , R. He , Z. Yang , X. Peng , S. Hu , G. Yang , Genomics Proteomics Bioinformatics 2022, 20, 366. 34487863 10.1016/j.gpb.2021.08.002 PMC9684166

[146] P. Davis , M. Zarowiecki , V. Arnaboldi , A. Becerra , S. Cain , J. Chan , W. J. Chen , J. Cho , E. da Veiga Beltrame , S. Diamantakis , S. Gao , D. Grigoriadis , C. A. Grove , T. W. Harris , R. Kishore , T. Le , R. Y. N. Lee , M. Luypaert , H.‐M. Müller , C. Nakamura , P. Nuin , M. Paulini , M. Quinton‐Tulloch , D. Raciti , F. H. Rodgers , M. Russell , G. Schindelman , A. Singh , T. Stickland , K. Van Auken , et al., Genetics 2022, 220, iyac003.35134929

[147] A. Gaulton , A. Hersey , M. Nowotka , A. P. Bento , J. Chambers , D. Mendez , P. Mutowo , F. Atkinson , L. J. Bellis , E. Cibrián‐Uhalte , M. Davies , N. Dedman , A. Karlsson , M. P. Magariños , J. P. Overington , G. Papadatos , I. Smit , A. R. Leach , Nucleic Acids Res. 2017, 45, D945. 27899562 10.1093/nar/gkw1074 PMC5210557

[148] C. Rustichelli , R. Avallone , A. Ferrari , Expert Opin. Pharmacother. 2022, 23, 653. 35319319 10.1080/14656566.2022.2057221

[149] P. Norman , P. Leeson , X. Rabasseda , J. Castaner , R. Castaner , Drugs Future 2000, 25, 1121.

[150] J. L. Arbuckle , Amos Dev. Corp. SPSS Inc 2011, 11, 1.

[151] S. Zhou , S. Chen , L. Xia , J. Dong , Y. Liu , Q. Yang , L. Zhang , X. Ai , Aquaculture 2024, 584, 740640.

[152] A. Waterhouse , M. Bertoni , S. Bienert , G. Studer , G. Tauriello , R. Gumienny , F. T. Heer , T. A. P. de Beer , C. Rempfer , L. Bordoli , R. Lepore , T. Schwede , Nucleic Acids Res. 2018, 46, W296. 29788355 10.1093/nar/gky427 PMC6030848

[153] C. Knox , M. Wilson , C. M. Klinger , M. Franklin , E. Oler , A. Wilson , A. Pon , J. Cox , N. E. (Lucy) Chin , S. A. Strawbridge , M. Garcia‐Patino , R. Kruger , A. Sivakumaran , S. Sanford , R. Doshi , N. Khetarpal , O. Fatokun , D. Doucet , A. Zubkowski , D. Y. Rayat , H. Jackson , K. Harford , A. Anjum , M. Zakir , F. Wang , S. Tian , B. Lee , J. Liigand , H. Peters , R. Q. (Rachel) Wang , et al., Nucleic Acids Res. 2024, 52, D1265. 37953279 10.1093/nar/gkad976 PMC10767804

[154] S. Kim , J. Chen , T. Cheng , A. Gindulyte , J. He , S. He , Q. Li , B. A. Shoemaker , P. A. Thiessen , B. Yu , L. Zaslavsky , J. Zhang , E. E. Bolton , Nucleic Acids Res. 2025, 53, D1516. 39558165 10.1093/nar/gkae1059 PMC11701573

[155] N. M. O'Boyle , M. Banck , C. A. James , C. Morley , T. Vandermeersch , G. R. Hutchison , J. Cheminformatics 2011, 3, 33. 10.1186/1758-2946-3-33 PMC3198950 21982300

[156] S. Dallakyan , A. J. Olson , in Chemistry Biology Methods Protocol, (Eds.: J. E. Hempel , C. H. Williams , C. C. Hong ), Springer, New York, NY, pp. 243.

[157] O. Trott , A. J. Olson , J. Comput. Chem. 2010, 31, 455. 19499576 10.1002/jcc.21334 PMC3041641

[158] D. Seeliger , B. L. de Groot , J. Comput. Aided Mol. Des. 2010, 24, 417. 20401516 10.1007/s10822-010-9352-6 PMC2881210

[159] Y. M. Bradford , C. E. Van Slyke , L. Ruzicka , A. Singer , A. Eagle , D. Fashena , D. G. Howe , K. Frazer , R. Martin , H. Paddock , C. Pich , S. Ramachandran , M. Westerfield , Genetics 2022, 220, iyac016. 35166825 10.1093/genetics/iyac016 PMC8982015

[160] V. Thumuluri , J. J. Almagro Armenteros , A. R. Johansen , H. Nielsen , O. Winther , Nucleic Acids Res. 2022, 50, W228. 35489069 10.1093/nar/gkac278 PMC9252801

[161] O. Emanuelsson , S. Brunak , G. von Heijne , H. Nielsen , Nat. Protoc. 2007, 2, 953. 17446895 10.1038/nprot.2007.131

[162] J. D. Bendtsen , L. J. Jensen , N. Blom , G. von Heijne , S. Brunak , Protein Eng. Des. Sel. 2004, 17, 349. 15115854 10.1093/protein/gzh037

[163] J. J. Almagro Armenteros , K. D. Tsirigos , C. K. Sønderby , T. N. Petersen , O. Winther , S. Brunak , G. von Heijne , H. Nielsen , Nat. Biotechnol. 2019, 37, 420. 30778233 10.1038/s41587-019-0036-z

[164] A. Krogh , B. Larsson , G. von Heijne , E. L. L. Sonnhammer , J. Mol. Biol. 2001, 305, 567. 11152613 10.1006/jmbi.2000.4315

[165] M. E. Smoot , K. Ono , J. Ruscheinski , P.‐L. Wang , T. Ideker , Bioinformatics 2011, 27, 431. 21149340 10.1093/bioinformatics/btq675 PMC3031041

[166] A. Hagberg , P. Swart , *Proc. Python Sci. Conf*. California 2008.

[167] G. Yu , L.‐G. Wang , Y. Han , Q.‐Y. He , OMICS J. Integr. Biol. 2012, 16, 284. 10.1089/omi.2011.0118 PMC3339379 22455463

