# Supplementary material for: Genome‐Wide Protein Interaction Analysis in Parasitic Gyrodactylus Flatworms–Fish Hosts System and Drug Target Identification
Source: Adv Sci (Weinh). 2025 Sep 29;12(45):e14618. doi: 10.1002/advs.202514618 (PMC12677651; doi:10.1002/advs.202514618)
Supplement: Supplementary file 1 — Supporting Information [file ADVS-12-e14618-s001.pdf]

Supplementary file 1

for

Genome-Wide Protein Interaction Analysis in Parasitic Gyrodactylus Flatworms - Fish Hosts System and Drug Target Identification

*Dong Zhang, Jie-Mei Zhao, Chuan-Yu Xiang, Yi-Wen Ma, Hong-Peng Lei, Yu-Ying Shi, Shun Zhou, Xiaofei Zeng, Jin-Song Chen, Fei Liu, Ben-He Zeng, Ye Hu, Rui Song, Feng Zhang, Xiang Liu, Wen-Xiang Li, Gui-Tang Wang, Ivan Jakovlić**

* Corresponding author: Ivan Jakovlić, State Key Laboratory of Herbage Improvement and Grassland Agro-ecosystems, and College of Ecology, Lanzhou University, 730000, Lanzhou, China; Phone: +86-18571472074; Email: jakovlici@lzu.edu.cn and ivanjakovlic@yahoo.com

Table S1. The total number of repeat elements identified in the two haplotypes of *G. kobayashii* and the other two gyrodactylids using the Extensive de-novo TE Annotator (EDTA) pipeline. LTR (long terminal repeats); Gypsy - a type of retrotransposon, a mobile genetic element found in various organisms; TIR: terminal inverted repeat; Mutator: a genetic element or mutation that plays a significant role in cellular genetic variation; PIF_Harbinger, Tc1_Mariner, hAT and helitron: four types of transposable elements. For *G. kobayashii* haplotypes, the total size in Mb and the proportion of the total genome (%) are also shown in brackets.

Species	*G. kobayashii* (hap1/hap2)	*G. bullatarudis*	*G. salaris*
LTR	--	--	--
Gypsy	6188 (2.98Mb; 3.4%)/8549 (3.6Mb; 3.95%)	5395	17
unknown	1134 (0.39Mb; 0.44%)/4519 (2Mb; 2.20%)	21392	8
TIR	--	--	--
CACTA	3311 (0.77Mb; 0.88%)/5489 (1.11Mb; 1.22%)	7071	3364
Mutator	26769 (13.56Mb; 15.49%)/29873 (18.67Mb; 20.48%)	23594	14255
PIF_Harbinger	2477 (0.54Mb; 0.62%)/267 (0.06Mb; 0.06%)	1121	292
Tc1_Mariner	21 (27372bp; 0.03%)/15 (14101bb; 0.02%)	2582	1380
hAT	5327 (1.34Mb; 1.53%)/3756 (1.02Mb; 1.12%)	7991	10699
nonTIR	--	--	--
helitron	3245 (0.61Mb; 0.70%)/1658 (0.33Mb; 0.36%)	842	9758
Total	48472 (20.23Mb; 23.09%)/54126 (26.81Mb; 29.42%)	69988	39773

Table S2. The identification of repeated sequences in the two haplotypes (hap1/hap2 respectively) of *G. kobayashii* genome using TRF, RepeatMasker, and RepeatProteinMask tools.

Type	Repeat Size(bp)	% of genome
TRF	20348/21512	0.0023/0.024
RepeatMasker	21183719/7810647	24.18/8.57
RepeatProteinMask	3311330/3703721	3.80/4.06

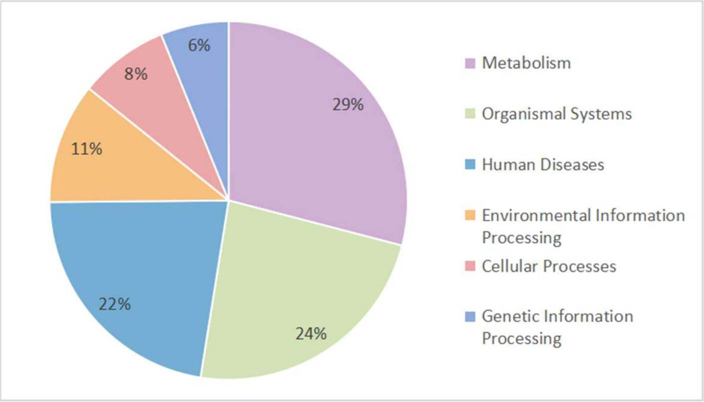

Figure S1. Pie chart showing the distribution of annotated hap1 genes in 358 KEGG biological pathways.

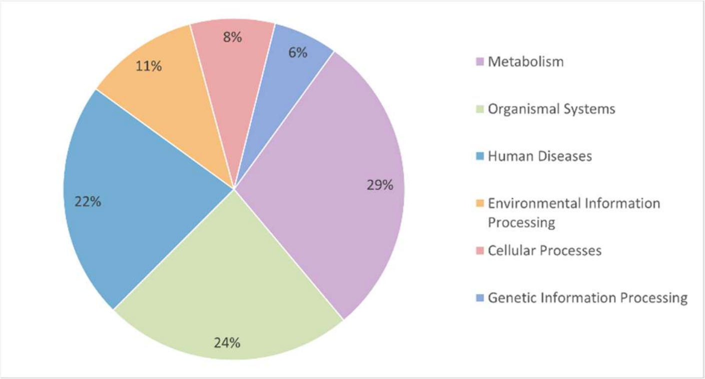

Figure S2. Pie chart showing the distribution of annotated hap2 genes in 360 KEGG biological pathways.

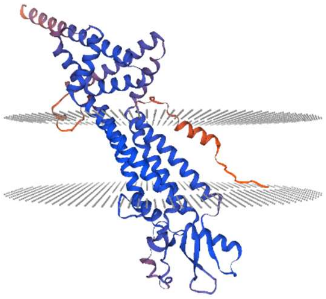

Figure S3. Predicted tertiary structure of *G. kobayashii* innexin protein generated by homology modeling through SWISS-MODEL. The model was constructed using *Taenia asiatica* (UniProt ID: A0A158R9B3) as the template. Model quality (0.33) was assessed by GMQE. Secondary structure elements are shown as ribbons: α-helices in red, β-sheets in blue.

Table S3. Key parameters of the top 30 virtual drug screening hits. DB ID is DrugBank ID, BA is Binding affinity in kcal/mol, Weight is molecular weight in KDa, and R-5 indicates compliance with Lipinski's "rule of five" (MW ≤500, LogP ≤5, HBD ≤5, HBA ≤10).

DB ID	BA	Name	Formula	Weight	R-5
DB16098	-9.2	Atogepant	$C_{29}H_{23}F_6N_5O_3$	603.52	Yes
DB15328	-9.1	Ubrogepant	$C_{29}H_{26}F_3N_5O_3$	549.5	Yes
DB00320	-9.0	Dihydroergotamine	$C_{33}H_{37}N_5O_5$	679.79	No
DB11986	-8.7	Entrectinib	$C_{31}H_{34}F_2N_6O_2$	560.64	No
DB00872	-8.5	Conivaptan	$C_{32}H_{26}N_4O_2$	498.57	No
DB15688	-8.5	Zavegepant	$C_{36}H_{46}N_8O_3$	638.80	No
DB14989	-8.4	Umbralisib	$C_{31}H_{24}F_3N_5O_3$	571.55	No
DB00966	-8.3	Telmisartan	$C_{33}H_{30}N_4O_2$	514.62	No
DB08827	-8.2	Lomitapide	$C_{39}H_{37}F_6N_3O_2$	693.72	No
DB09280	-8.1	Lumacaftor	$C_{24}H_{18}F_2N_2O_5$	452.41	Yes
DB15011	-8.1	Avacopan	$C_{33}H_{35}F_4N_3O_2$	581.64	Yes
DB06595	-8.1	Midostaurin	$C_{35}H_{30}N_4O_4$	570.64	No
DB01126	-8.1	Dutasteride	$C_{27}H_{30}F_6N_2O_2$	528.53	No
DB01336	-8.0	Metocurine	$C_{40}H_{48}N_2O_6$	652.8	No
DB00762	-8.0	Irinotecan	$C_{33}H_{38}N_4O_6$	586.68	No
DB08881	-8.0	Vemurafenib	$C_{23}H_{18}ClF_2N_3O_3S$	489.92	No
DB12457	-7.9	Rimegepant	$C_{28}H_{28}F_2N_6O_3$	534.56	No
DB11262	-7.9	Bisoctrizole	$C_{41}H_{50}N_6O_2$	658.87	No
DB14895	-7.9	Vibegron	$C_{26}H_{28}N_4O_3$	444.53	No
DB14703	-7.9	Dexamethasone metasulfobenzoate	$C_{28}H_{30}FNaO_9S$	584.59	No
DB01117	-7.9	Atovaquone	$C_{22}H_{19}ClO_3$	366.84	No
DB01199	-7.9	Tubocurarine	$C_{37}H_{41}N_2O_6$	609.73	No
DB04835	-7.9	Maraviroc	$C_{29}H_{41}F_2N_5O$	513.67	No
DB00210	-7.9	Adapalene	$C_{28}H_{28}O_3$	412.52	No
DB00288	-7.8	Amcinonide	$C_{28}H_{35}FO_7$	502.57	No
DB11262	-7.8	Bisoctrizole	$C_{41}H_{50}N_6O_2$	658.87	No
DB00619	-7.8	Imatinib	$C_{29}H_{31}N_7O$	493.60	Yes
DB11791	-7.8	Capmatinib	$C_{23}H_{17}FN_6O$	412.42	Yes
DB00549	-7.8	Zafirlukast	$C_{31}H_{33}N_3O_6S$	575.68	No
DB00496	-7.8	Darifenacin	$C_{28}H_{30}N_2O_2$	426.55	Yes

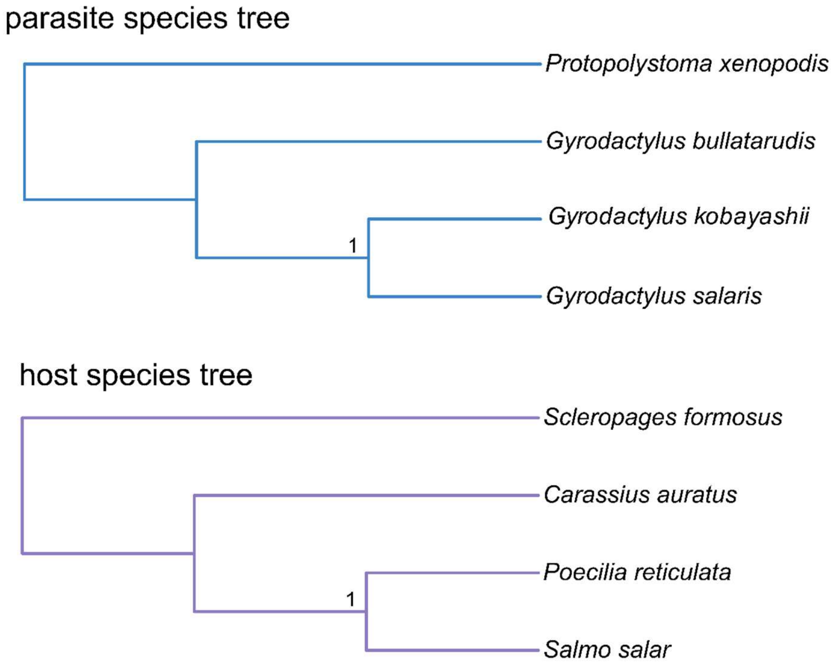

Figure S4. Confirmation of species tree topologies for parasites and hosts inferred using ASTRAL and concatenated 1040 and 379 single-copy orthologues respectively. *Protopolystoma xenopodis* and *Scleropages formosus* are outgroups. Support for nodes is shown next to them.

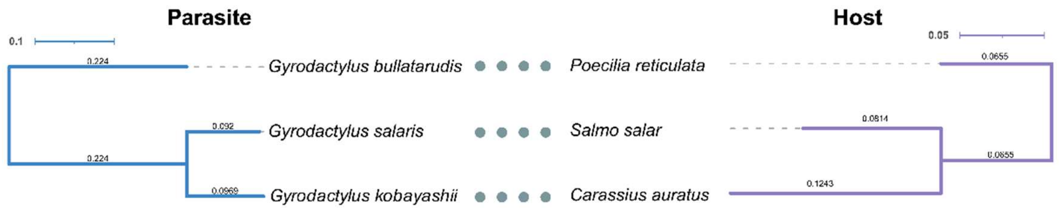

Figure S5. Phylogenies inferred using concatenated genes identified in parasite and host PPI datasets. Branch lengths are shown on the branches. Please refer to Figure 3 in the main manuscript for corresponding species phylogenies.

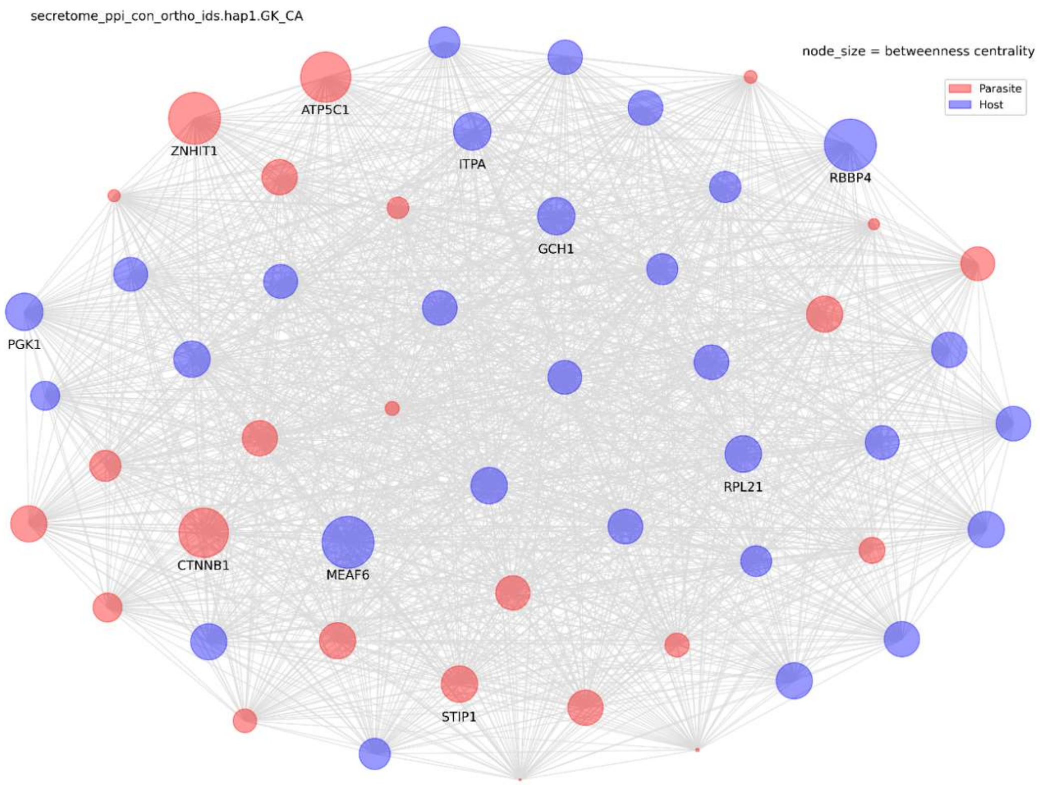

Figure S6. PPI network analysis showing the top 10% of proteins identified in the interactome between *Gyrodactylus kobayashii* and *Carassius auratus* ranked by betweenness centrality before applying the phylogenetic congruence filter. The node size is positively correlated to the betweenness centrality value, and node colour corresponds to the host/parasite side of the PPI (see legend in the figure). Protein names are shown for the top-ten ranked nodes.

Sequences producing significant alignments Download Select columns Show 100

select all *100 sequences selected* GenBank Graphics Distance tree of results MSA Viewer

Description	Scientific Name	Max Score	Total Score	Query Cover	E value	Per. Ident	Acc. Len	Accession
Gyrodactylus kobayashii isolate UK 18S ribosomal RNA gene, partial sequence; internal transcribed spacer 1, 5.8...	Gyrodactylus kob...	2187	2187	100%	0.0	100.00%	1226	AF484534.1
Gyrodactylus kobayashii strain GZ2 18S ribosomal RNA gene, partial sequence; internal transcribed spacer 1, 5....	Gyrodactylus kob...	2187	2187	100%	0.0	100.00%	1294	KC922452.1
Gyrodactylus kobayashii 18S rRNA gene (partial), 5.8S rRNA gene, 28S rRNA gene (partial) and internal transcri...	Gyrodactylus kob...	2176	2176	100%	0.0	99.83%	1243	AJ132985.3
Gyrodactylus kobayashii isolate HN 18S ribosomal RNA gene, partial sequence; internal transcribed spacer 1, 5.8...	Gyrodactylus kob...	2176	2176	100%	0.0	99.83%	1275	KJ524572.1
Gyrodactylus kobayashii strain CIFRI.GK-1 internal transcribed spacer 1, partial sequence; 5.8S ribosomal RNA g...	Gyrodactylus kob...	2159	2159	100%	0.0	99.58%	1297	PP922389.1
Gyrodactylus kobayashii isolate jsjy3 internal transcribed spacer 1, partial sequence; 5.8S ribosomal RNA gene, c...	Gyrodactylus kob...	2080	2080	95%	0.0	100.00%	1126	ON117569.1
Gyrodactylus kobayashii isolate HN1 18S ribosomal RNA gene, partial sequence; internal transcribed spacer 1, 5....	Gyrodactylus kob...	2067	2067	95%	0.0	99.82%	1255	KJ755085.1
Gyrodactylus kobayashii isolate HN2 18S ribosomal RNA gene, partial sequence; internal transcribed spacer 1, 5....	Gyrodactylus kob...	2028	2028	92%	0.0	100.00%	1247	KJ755086.1
Gyrodactylus sprostonae isolate GV7 internal transcribed spacer 1, partial sequence; 5.8S ribosomal RNA gene, ...	Gyrodactylus spr...	1192	1192	77%	0.0	90.41%	1150	OQ685907.1
Gyrodactylus sprostonae isolate GV6 internal transcribed spacer 1, partial sequence; 5.8S ribosomal RNA gene, ...	Gyrodactylus spr...	1192	1192	77%	0.0	90.41%	1141	OQ685906.1
Gyrodactylus sprostonae isolate GV9 internal transcribed spacer 1, partial sequence; 5.8S ribosomal RNA gene, ...	Gyrodactylus spr...	1192	1192	77%	0.0	90.41%	1149	OQ685909.1
Gyrodactylus sprostonae isolate GV10 internal transcribed spacer 1, partial sequence; 5.8S ribosomal RNA gene,...	Gyrodactylus spr...	1192	1192	77%	0.0	90.41%	1163	OQ685910.1
Gyrodactylus sprostonae isolate GV1 internal transcribed spacer 1, partial sequence; 5.8S ribosomal RNA gene, ...	Gyrodactylus spr...	1186	1186	77%	0.0	90.31%	1066	OQ685901.1
Gyrodactylus sprostonae isolate GV2 internal transcribed spacer 1, partial sequence; 5.8S ribosomal RNA gene, ...	Gyrodactylus spr...	1186	1186	77%	0.0	90.31%	1067	OQ685902.1

Figure S7. **Top BLAST hits for the *18S* sequence of *Gyrodactylus kobayashii*.**

Text S1. Transcriptome sequencing and assembly

The total RNA was extracted using TRIzol Reagent (Invitrogen, USA) following the manufacturer's protocol. Library construction was conducted by the Majorbio company (China) using TruseqTM RNA Sample Preparation Kit (Illumina, USA) and sequencing to generate 6G 150 bp paired-end reads per library on the Illumina HiSeq 4000 platform. The quality of the resulting reads was checked with FastQC (https://www.bibsonomy.org/bibtex/2b6052877491828ab53d3449be9b293b3), and low-quality bases and Illumina adapters were trimmed using FASTP (Chen et al. 2018). The remaining reads were assembled using Trinity (Grabherr et al. 2011), followed by the open reading frame (ORF) prediction and translation in TransDecoder (https://github.com/TransDecoder).

References

Chen, Shifu, Yanqing Zhou, Yaru Chen, and Jia Gu. 2018. 'Fastp: An Ultra-Fast All-in-One FASTQ Preprocessor'. *Bioinformatics* 34 (17): i884–90. https://doi.org/10.1093/bioinformatics/bty560.

Grabherr, Manfred G, Brian J Haas, Moran Yassour, et al. 2011. 'Full-Length Transcriptome Assembly from RNA-Seq Data without a Reference Genome'. *Nature Biotechnology* 29 (7): 644–52. https://doi.org/10.1038/nbt.1883.

Hahn, Christoph, Bastian Fromm, and Lutz Bachmann. 2014. 'Comparative Genomics of Flatworms (Platyhelminthes) Reveals Shared Genomic Features of Ecto- and Endoparastic Neodermata'. *Genome Biology and Evolution* 6 (5): 1105–17. https://doi.org/10.1093/gbe/evu078.

Romiguier, J., P. Gayral, M. Ballenghien, et al. 2014. 'Comparative Population Genomics in Animals Uncovers the Determinants of Genetic Diversity'. *Nature* 515 (7526): 261–63. https://doi.org/10.1038/nature13685.

Salte, Ragnar, Hans Bernhard Bentsen, Thomas Moen, et al. 2010. 'Prospects for a Genetic Management Strategy to Control Gyrodactylus Salaris Infection in Wild Atlantic Salmon (Salmo Salar) Stocks'. *Canadian Journal of Fisheries and Aquatic Sciences* 67 (1): 121–29. https://doi.org/10.1139/F09-168.
